# Skeletons in confusion: a review of astrophorid sponges with (dicho–)calthrops as structural megascleres (Porifera, Demospongiae, Astrophorida)

**DOI:** 10.3897/zookeys.68.729

**Published:** 2010-11-16

**Authors:** Rob W.M. Van Soest, Elly J. Beglinger, Nicole J. De Voogd

**Affiliations:** 1 Netherlands Centre for Biodiversity, Zoological Museum of Amsterdam, P.O. Box 94766, 1090 GT Amsterdam, The Netherlands; 2 National Centre for Biodiversity, Naturalis, P.O. Box 9517, 2300 RA Leiden, The Netherlands

**Keywords:** Sponges, Astrophorida, Pachastrellidae, *Dercitus*, Calthropellidae, *Calthropella*, taxonomy, new species

## Abstract

We present a review of astrophorid species possessing calthrops megascleres as structural megascleres (including species with dichotriaene modifications, but excluding mesotriaene and trichotriaene bearing species). Radiating oxeas characteristic of most astrophorids are lacking in such sponges, but auxiliary oxeas are apparently present in some species. These sponges are currently assigned to two families, Pachastrellidae with four nominal genera Dercitus, Stoeba, Dercitancorina, Halinastra (the latter two generally considered junior synonyms of Stoeba), and Calthropellidae with nominal genera Calthropella, Corticellopsis (usually considered a junior synonym), Pachataxa and Pachastrissa. Our review of many original specimens and extensive new material demonstrates the existence of considerable morphological similarity in megasclere shape and arrangement, and possible overlap of some microscleres of these sponges: pseudasters in Dercitus resembling euasters in Calthropella, ataxasters and other modified types in Calthropellidae resembling rhabds in a species of Dercitus. Pachastrellid representatives are proposed to be united in a single genus Dercitus, recognizable as (dicho–)calthrops bearing sponges with sanidaster–like microrhabds. Three subgenera, Dercitus s.s., Stoeba and the revived Halinastra are distinguished to accommodate species groups sharing additional characters. Dercitancorina is merged with Dercitus (Stoeba), because the type species, Dercitus lesinensis was found to be barely distinct from Dercitus (Stoeba) plicatus. Similarly, the calthropellid representatives are proposed to be united in a single genus Calthropella recognizable as calthrops bearing sponges with oxyasters. Three subgenera, Calthropella s.s., Pachataxa and Corticellopsis are distinguished to accommodate species groups sharing additional characters. The calthropellid genus Pachastrissa is considered a junior synonym of Calthropella s.s. because its type species, Pachastrella geodioides, is barely distinct from the type species of Calthropella, Calthropella simplex. Two species previously assigned to Dercitus or Stoeba (Dercitus loricatus and Stoeba natalensis) are excluded from the genus Dercitus as they do not fit with the emended and improved definition of the genus. One species assigned to Calthropella, Calthropella digitata, is excluded from that genus and assigned to the ancorinid genus Stelletta. Based on the similarity of the megascleres and their structure-less arrangement, we predict that the two reviewed genera will be found to be closely related and eventually will need to be accomodated in a single family, but independent molecular evidence is awaited before changes in the family classification, including a verdict on the validity of the family Calthropellidae, will be proposed. Our review included 38 valid species among which fourteen species and one subspecies appear to be new to science. Four of these were represented by insufficient material for a proper description, but the remaining ten are described as new species: Dercitus (Stoeba) senegalensis **sp. n.**, Dercitus (Stoeba) verdensis **sp. n.**, Dercitus (Stoeba) fijiensis **sp. n.**, Dercitus (Stoeba) bahamensis **sp. n.**, Dercitus (Halinastra) berau **sp. n.**, Dercitus (Halinastra) japonensis **sp. n.**, Dercitus (Halinastra) arubensis **sp. n.**, Dercitus (Halinastra) sibogae **sp. n.**, Calthropella (Calthropella) xavierae **sp. n.**, and Calthropella (Pachataxa) pyrifera **sp. n.** The new subspecies, Dercitus (Dercitus) bucklandi lusitanicus **ssp. n.** is described for southern East Atlantic populations of the nominal species. Several specimens assigned to existing species were found to be deviating to the extent that they are likely members of further undescribed species. These are briefly discussed to facilitate further studies of the diversity of the two genera. Species of both genera and the six subgenera, including deviating or insufficiently characterized specimens belonging to species not yet properly described, are keyed out. Distribution patterns are discussed.

## Introduction

The present study addresses the taxonomy of some genera of the order Astrophorida (Dercitus Gray (1867) and Calthropella Sollas (1888) and their relatives), that appear or seem to share characters which do not justify their present allocation in different families. The main character shared is the structural position of calthrops megascleres (and derivates thereof) and the absence of structural oxea megascleres. Skeletons built from that megasclere complement lack a radiate structure making member species thickly or massively encrusting or insinuating. Such sponges are easily missed in trawl or dredge surveys and their diversity appears underestimated. Below, known members of these taxa will be reviewed and new members will be described and extensively illustrated.

A further purpose of this paper is to propose a slightly altered generic allocation of species, facilitating easier recognition of the morphological classification, but the familial allocations will be left as they are in the Systema Porifera (Hooper and Van Soest 2002) until such time that sufficient molecular systematic investigations have been made for a reshuffling of genera and families in the Astrophorida. Such studies are well on the way (Cárdenas 2010; Cárdenas et al. 2010), but face considerable problems of DNA extraction, especially from old type material, making the present study a timely contribution to astrophorid systematics. Before entering the systematic descriptions we present an overview of the current status of the target genera to explain why the generic allocations need to be improved. For explanation of the terms for spicules and other skeletal features the reader is referred to the Thesaurus of Sponge Morphology (Boury-Esnault and Rützler 1997), which may be freely downloaded from the World Porifera Database (Van Soest et al. 2008).

## Astrophorida possessing calthrops megascleres as structural spiculation

### Astrophorida

Astrophorida Sollas (1888) is a well-defined order of Demospongiae comprising sponges with the combination of triaene megascleres and aster microscleres, usually complemented with large oxea megascleres. The oxeas and long-shafted triaenes are arranged perpendicularly to the surface providing a radiate structure – at least in peripheral regions - which is shared with members of the order Spirophorida (Hooper and Van Soest 2002). Astrophorida and Spirophorida show a clear overlap with ‘Lithistida’ and future studies will undoubtedly demonstrate that this polyphyletic assemblage will have to be subdivided over these two and possibly other orders. Notwithstanding a general agreement over the non-lithistid Astrophorida as a well-founded ordinal taxon, family group distinction within this order remains tentative, with five families currently defined, showing overlapping morphologies and lacking proper synapomorphies (Cárdenas 2010; Cárdenas et al. 2010), instead of which they are loosely defined on combinations of characters. Two such families seemingly showing overlap in spicular characters are Pachastrellidae Carter (1875) and Calthropellidae Lendenfeld (1907), as recently redefined by respectively Maldonado (2002) and Van Soest and Hooper (2002). Both families encompass a few genera lacking the usual structural oxeas and long-shafted triaenes which prevail in most Astrophorida, although persistent reports for the occurrence of auxiliary or accessory oxeas of variable sizes are found in the descriptions of several species of these genera (see below).

### Pachastrellidae and Calthropellidae

The Pachastrellidae genera Dercitus Gray (1867), Stoeba Dendy (1905), with alleged synonyms Dercitancorina Topsent (1902) and Halinastra de Laubenfels (1936) (see Maldonado 2002), share the possession of exclusively (dicho-)calthrops megascleres (or short-shafted (dicho-)triaenes) and confusedly arranged skeleton with two genera of the family Calthropellidae, Calthropella Sollas (1888) (with alleged synonym Corticellopsis Bergquist, 1968) and Pachataxa de Laubenfels (1936). The pachastrellid members of this paraphyletic assemblage possess irregular acanthomicrorhabd-like sanidasters, but lack asterose microscleres, the calthropellid members possess euaster microscleres, lacking sanidasters. Calthrops megascleres or short-shafted triaenes and their dichotriaene modifications have a wide but infrequent distribution over species of various tetractinellid genera, e.g. Paratetilla Dendy (1905) (Spirophorida: Tetillidae), Erylus Gray (1867), Caminus Schmidt (1862) (Astrophorida: Geodiidae), Penares Gray (1867), Stryphnus Sollas (1886) (Astrophorida: Ancorinidae), Pachastrella Schmidt (1868), Triptolemma de Laubenfels (1955) (Astrophorida: Pachastrellidae), but in most cases – with some exceptions in Pachastrella – these are accompanied by oxeas as structural megascleres, and generally the structure of the skeleton is radiate or sub-radiate (Hooper and Van Soest 2002). In pachastrellid Dercitus and Stoeba and calthropellid Calthropella and Pachataxa we find calthrops as the only megasclere type.

### Dercitus and its relatives

The monospecific genus Dercitus (for Dercitus bucklandi (Bowerbank, 1858) and its closely related ‘sister’genus Stoeba are uncharacteristic Pachastrellidae, with none of the usual streptasters, long-shafted triaenes and long oxeas found in the majority of the genera. The acanthomicrorhabd microscleres are usually considered derivates of sanidasters, and will be further called as such below, but they are characteristically irregular and variable. Small incipient sanidasters appear to be microxea-like with only occasional spines. In addition to these microscleres, Dercitus possesses toxa-like spicules, which are lacking in Stoeba. This is the only known difference between these ‘genera’. Many authors prior to the appearance of the Systema Porifera did not distinguish between Stoeba and Dercitus, because the occurrence of toxas in Dercitus bucklandi was not considered of generic value. There is only a single species with this unique character, and defining separate genera Dercitus and Stoeba is not possible without referring to the absence or presence of this same unique character (so called ‘A-not A’ classification). Dercitus and Stoeba possess this limited set of calthrops and (dicho-)calthrops megasclere spicules, but some species allegedly have a complement of (rare) oxeas. For such species, the genus Dercitancorina was erected by Topsent (1902), type Pachastrella lesinensis Lendenfeld (1894), but the reexamination of the type specimen of Pachastrella lesinensis did not reveal the presence of oxeas (see below). Possession of oxeas in species of this group is controversial against the possibility that the often broken and variously sized, ‘auxiliary’ megascleres may be contaminations. A further genus Halinastra de Laubenfels (1936) was erected for Dercitus exostoticus (Schmidt 1868 as Pachastrella exostotica) which has peculiar aster-like microscleres hypothesized to be compressed sanidasters (Maldonado 2002). It was synonymized with Stoeba by Maldonado (l.c.), but we demonstrate here that several more such species with ‘compressed’ sanidasters exist, making resurrection at the subgenus level a logical consequence. In summary, we propose to employ a modified classification of Dercitus s.l. into three subgenera: Dercitus (Dercitus), Dercitus (Stoeba) and Dercitus (Halinastra).

### Calthropella and its relatives

Calthropellidae (with type genus Calthropella) as recently defined have only four recognized genera (Van Soest and Hooper 2002), but there is considerable confusion over the generic definitions, which may possibly overlap. At least one genus, Chelotropella Lendenfeld (1907), appears misapplied as a member of Calthropellidae because the family is based on the possession of short-shafted triaenes in combination with euasters, while Chelotropella has long-shafted triaenes and in combination with oxeas forms a radiate skeleton similar to genera of the Ancorinidae such as Stelletta. A further genus that is in dispute is Pachastrissa Lendenfeld (1903), based on a type species, Pachastrella geodioides Carter (1876), that seems to differ from the type species of Calthropella (Calthropella simplex Sollas, 1888) only in the alleged presence of proper oxeas in addition to habit, skeletal structure and spiculation identical to that of Calthropella simplex. A fourth genus, Pachataxa de Laubenfels (1936) will be demonstrated below to be so close to Calthropella that it is proposed to merge it with that genus. It is defined as having in addition to euasters peculiar irregular microspined silica-bodies, called ataxasters. SEM studies of these ataxasters demonstrate that they are not far removed from hypersilicified aster-derived microscleres found in several Calthropella species. Corticellopsis Bergquist (1968) was erected to replace the preoccupied Corticella Sollas (1888) with type species Corticium stelligerum Schmidt (1868) and subsequently synonymized with Calthropella by Van Soest and Hooper (2002) because of similarity of spicule complement (calthrops and euasters). However, this similarity is mostly on paper as its euasters differ clearly in shape from those of Calthropella simplex and Calthropella geodioides: the former has spherasters with tuberculate rays, while the latter has commonplace strongylasters. As a consequence of these observations, the calthropellid genera Calthropella and Pachataxa are proposed to be recognized only at the subgenus level, to which the formerly synonymized Corticellopsis is joined as a third subgenus, resulting in the taxa Calthropella (Calthropella), Calthropella (Pachataxa) and Calthropella (Corticellopsis).

### Similarities of Dercitus and Calthropella

The calthropellid Calthropella (Pachataxa) with peculiar ataxaster microscleres and several species of the pachastrellid genus Dercitus with compressed aster-like sanidasters or smooth or faintly acanthose microrhabds (see below) appear to some extent to bridge the gap between the two family groups. Euasters of Calthropella (Pachataxa) appear peculiar in having a thick centre and numerous short and irregular rays, whereas the ataxasters are often malformed and irregularly spined, occasionally smooth.

Dercitus and its relatives (including Stoeba, Dercitancorina and Halinastra)could be envisaged to reside more comfortably in the same family as Calthropella as a genus devoid of true euasters, but otherwise similar to other genera of that family. Current assignment to Pachastrellidae is not clearly warranted by pachastrellid apomorphies, as the microsclere complement of sanidasters is of astrophorid-wide occurrence including the Ancorinidae. We pose here the question, whether there is sufficient evidence to suggest these genera should be united within the same family. Ultimately, such a decision should be based on independent molecular and morphological evidence, so we refrain from making formal proposals for change and for the time being the family assignment of the genera is left as it appears in the Systema Porifera (Maldonado 2002; Van Soest and Hooper 2002).

### Triptolemma and Thrombidae

We will not include in our study the pachastrellid genus Triptolemma de Laubenfels (1955) and the thrombid genera Thrombus Sollas (1886) and Yucatania Gómez (2006), despite the fact that these genera all have short-shafted triaenes and lack proper radiating oxeas. Triptolemma is similar to Dercitus and Calthropella in having calthrops in a large size range and the insinuating habit conforms to that of some species of Dercitus. It is not considered very likely that Triptolemma is closely related as it has a dominance of mesotriaene (dicho-)calthrops and its microsclere complement includes true streptasters (amphiasters, spirasters). Thrombus and Yucatania have their (tricho-)triaenes entirely spined and microscleres are peculiar birotule-like amphiasters.

### ‘Phantom’ oxeas

Several species of Dercitus s.l. and Calthropella s.l. have been persistently attributed with the possession of long oxeas as part of the megasclere complement. The report of smaller or longer diactines (oxeas, strongyles) in species of Dercitus (Stoeba) is a recurrent point of discussion. Authors either consider them proper but so far have failed to indicate a structural position of such spicules in the skeleton, or they are presumed contaminations. In favor of the latter point of view is that most Dercitus s.l. are either insinuating or consolidating, thus coming into close contact with sediment including loose spicules. Diactines are ubiquitous spicules, produced mostly by Haplosclerida and Halichondrida. Longer oxeas have frequently been mentioned in descriptions of Dercitus and Calthropella alike and such spicules are common in Astrophorida in general, so are more likely to be proper. Nevertheless, it is clear in most cases that they do not take up a structural position in the skeletal architecture, so if they are to be considered proper, they are at best ‘auxiliary’ and are here regarded as vestiges of their astrophorid ancestry. In many cases, authors expressly mention that the long oxeas are invariably broken or rare, which strengthens the assumption they are not proper.

Species with structural oxeas in a radial position are here excluded and referred to other astrophorid genera.

### Biogeography

A recent paper by Moraes and Muricy (2007), describing a new Stoeba from Brazilian waters, suggested in a world distribution map of species of Stoeba that records of the genus would be bsent from the Caribbean region. Strictly speaking this was true, but there were previous records of Dercitus sp. from various parts of the Caribbean (Kobluk and Van Soest 1989: Bonaire; Djura and Faulkner 1980 and Rützler et al. 2000: Belize; Kohmoto et al. 1988; Burres et al. 1989: Bahamas). The new classification of Dercitus s.l. (including Stoeba) will thus remove this distributional anomaly (lack of a circumtropical taxon from the Caribbean region). Similarly, the new assignments of calthropellid genera result in a continuous circumglobal distribution of Calthropella s.l. whereas this was formerly confined to an irregular circum-African pattern plus an outlier in New Zealand (Calthropella s.s.) and a disjunct Caribbean – New Caledonian occurrence (Pachataxa).

### Contents of the present study

Below, we review the species of the (sub-)genera Dercitus (Dercitus), Dercitus (Stoeba), Dercitus (Halinastra), Calthropella (Calthropella), Calthropella (Pachataxa) and Calthropella (Corticellopsis) based on lists obtained from the World Porifera Database (Van Soest et al. 2008).Although our paper has monographical ambitions the current state of our knowledge of these sponges forces us to refrain from making comprehensive descriptions of all extant species. Almost invariably, species delimitation becomes problematic when more than a single specimen of a nominal species has been described. For this reason, a fairly large number of suspected species remain unnamed and inadequately described, whereas some species suspected to be synonymous with others remain accepted. In addition, poor material of several species and difficulties in obtaining all relevant type specimens also contribute to this study failing to reach a full blown revision of Dercitus and Calthropella. We treat here a total of 38 species, 24 species of Dercitus s.l. (and one new subspecies), and 14 species of Calthropella s.l.

## Material and methods

Specimens in the collections of the National Centre for Biodiversity (formerly Zoological Museum of the University of Amsterdam (**ZMA**) and National Museum of Natural History, Naturalis (**RMNH**), Leiden) were obtained by various expeditions and individual collectors over a large period of time. Additionally, type and other specimens were borrowed from several institutions, including the Museum für Naturkunde, Berlin (**ZMB**), the Smithsonian Institution, Washington (**USNM**), Museum of Comparative Zoology, Harvard (**MCZ**), the Natural History Museum (London) (**BMNH**), the Muséum National d’Histoire Naturelle, Paris (**MNHN**), the Landesmuseum Graz, Austria (**LMJG**), the Zoological Museum of Copenhagen (**ZMUC**), the Harbor Branch Oceanographic Institution, Fort Pierce, USA (**HBOI**), and the Museu Nacional de Rio de Janeiro (**MNRJ**). Through the courtesy of Dr. Joana Xavier (Research Centre in Biodiversity and Genetic Resources, CIBIO, Department of Biology, University of the Azores) we were able to study specimens in her care from Portuguese shallow-water and deep sea localities. Full specimen data are provided with the description of the species.

Specimens were studied from thick sections cut at right angles to the surface and from dissociated spicules, using light microscopy and a JEOL Scanning Electron Microscope. Digital images of the spicules were assembled on a black background and aligned and cleaned up using Adobe Photoshop CS3.

The left over part of the spicule suspension was used for light microscopy measurements. Measurements of megascleres include for calthrops cladus length × width, cladome size, i.e. the distance between the apex of a cladus and the imaginary line between two opposing cladi endings; for dichocalthrops, protocladus length × width, deuterocladus length × width, rhabdus length × width and cladome size. Measurements of microscleres include for sanidasters length × width including the spines, for toxa-like microscleres length × width, for euasters greatest diameter including rays. Unless otherwise stated, minimum-*mean*-maximum from 25 of each spicule type encountered are given. To facilitate comparisons of cladome sizes of our own spicules and those from the literature, in case cladome sizes were not provided by the original authors these were artificially standardized by multiplying cladus length ×1.5 (calthrops) and the sum of protocladus and deuterocladus length ×1.9 (dichocalthrops), based on average ratios obtained from random measurements of various specimens. Such artificially standardized data are indicated by an asterisk.

In the descriptions and definitions, the word calthrops will be used in singular and plural sense (in accordance with Sollas 1888: lx). The Thesaurus of Sponge Morphology (Boury-Esnault and Rützler 1997) erroneously referred to the singular of this spicule as ‘calthrop’, possibly because Lendenfeld (1903) proposed ‘chelotrop’ as term for the same spicule. The dichotriaene modification will be termed dichocalthrops below.

## Key to Astrophorida possessing (dicho-)calthrops as structural megascleres (lacking structural oxeas and long-shafted triaenes)

**Table:** 

1	Megascleres include trichotriaenes	Thrombidae (not further treated here)
–	No trichotriaenes	2
2	Megascleres include meso-dichotriaenes; microscleres include streptasters	Triptolemma (not further treated here)
–	Megascleres do not include meso-dichotriaenes	3
3	Microscleres include streptasters (amphiasters, spirasters, metasters)	Pachastrellidae (not further treated here)
–	Microscleres sanidasters, ataxasters or euasters; no streptasters	4
4	Microscleres include sanidasters, no euasters	Dercitus p. 9
–	Microscleres include euasters in some form; no sanidasters	Calthropella p. 54

## Systematic Descriptions

Phylum Porifera

Class Demospongiae

Order Astrophorida

### Family Pachastrellidae

#### Genus Dercitus

Gray, 1867

Battersbyia Bowerbank 1874
Stoeba Sollas 1888
Calcabrina Sollas 1888
Halinastra de Laubenfels 1936
Dercitancorina Topsent 1902

##### Type species:

Halina bucklandi Bowerbank, 1858.

##### Definition (emended):

Pachastrellidae (?) with calthrops or dichocalthrops as megascleres and possessing irregular acanthomicrorhabd-like sanidasters with a thick central axis relative to the actines; further microscleres may include smooth toxa-like forms and aster-like compressed forms; no structural oxea megascleres.

##### Comments:

**Figure 1.**
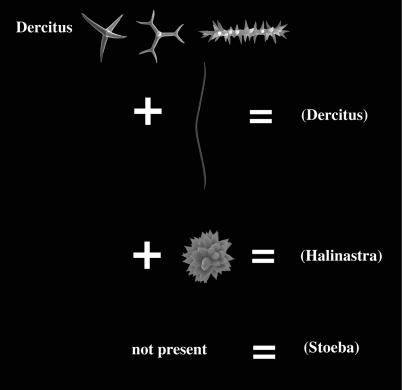
Key to the subgenera of the genus Dercitus.

Many authors have pointed out the similarities of Dercitus and Stoeba, the distinction of which rests entirely on the presence of unique toxa-like microscleres in the type species of the former. The similarities are the shape and size of the calthrops/short-shafted triaenes, the shape of the irregular sanidasters, the absence of any structure in the skeleton, the compressible liver-like texture, and the presence of large cells (60 µm) with inclusions reported for various Stoeba species as well as Dercitus bucklandi. The majority of past authors recognized only Dercitus, but Maldonado (2002) insisted on retaining the distinction at the genus level. Since there are dozens of species conforming to the above given definition and only a single North Atlantic species possesses the toxas, it appears unnecessarily formal to keep the use of two genus names for such similar species. It also would confound biogeographical analysis having a single unique character place an endemic genus in an area of the world (Celtic Seas and Lusitanian waters) where such higher taxa endemism in sponges is virtually nonexistent. Ultimately, we need independent molecular evidence to demonstrate that both are members of the same clade, but pending such results, we propose here for practical reasons to lower the status of the genera Dercitus and Stoeba to the level of subgenera. Dercitus being the senior name, the subgenera will be Dercitus (Dercitus) and Dercitus (Stoeba). A third subgenus proposed is the suppressed Halinastra de Laubenfels (1936). The subgenera will be defined below and are keyed out in Fig. 1.

Calthrops are characteristically variable in size (cladi measuring from 25 – 648 µm), and shape, with conical-straight, curved, stunted, deformed, bifid cladi, frequently one of the cladi being longer than the others, sometimes lacking one (‘tripods’) cladus or with one or more reduced cladi, occasionally with five or more cladi. Short-shafted dichotriaenes (dichocalthrops) are present in twelve of the currently named and recognized species, and in five of these have replaced the calthrops entirely. When both megasclere types are combined in a single species, dichocalthrops are often distinctly smaller than the calthrops and the proportion of the two varies among individuals of the species.

Natural products: three unrelated compounds with biological actitivity, such as anti-tumor or antibiotic activity, have been extracted from species of Dercitus, viz. methylaplysinopsins from a Belize specimen of Dercitus sp. (cf. Djura and Faulkner 1980) and the acridine alkaloid dercitine and a dimethyldihydroxylindoliniumchloride from Dercitus (Stoeba) bahamensis sp. n. (Gunawardana et al. 1988; Burres et al. 1989; Kohmoto et al. 2005 as Dercitus sp.). The furanosesterpene shinsonefuran was reported from Dercitus (Halinastra) japonensis sp. n. (Phuwapraisirisan et al. 2004, as Stoeba extensa).

##### Key to the subgenera of Dercitus (see Fig. 1).

**Table:** 

1	Toxa-like microscleres present	Dercitus (Dercitus)
–	No toxa-like microscleres	2
2	Sanidasters divisble in two categories, thin-and-long and thick-and-short, the latter often compressed to form pseudasters	Dercitus (Halinastra)
–	Sanidasters may be variable but not clearly divisible in shape categories, no pseudasters	Dercitus (Stoeba)

##### Subgenus Dercitus

Gray, 1867

###### Definition:

Dercitus possessing toxa-like microscleres and a single category of irregular sanidasters.

###### Type species:

Halina bucklandi Bowerbank, 1858

###### Key to the taxa of Dercitus (Dercitus).

**Table:** 

1	Robust toxas, up to 100 µm in length	Dercitus (Dercitus) bucklandi subsp. bucklandi
–	Thin toxas only up to 70 µm in length	Dercitus (Dercitus) bucklandi subsp. lusitanicus ssp.n.

###### Dercitus (Dercitus) bucklandi subsp. bucklandi

(Bowerbank, 1858)

**Figure 2.**
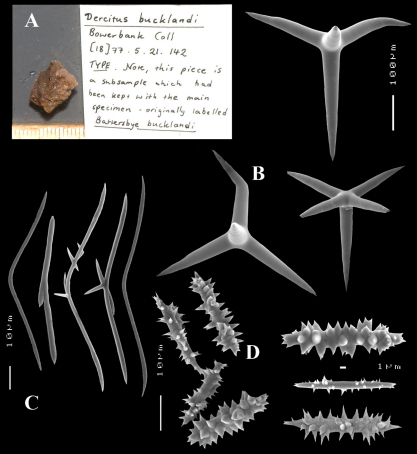
Dercitus (Dercitus) bucklandi, fragment of type BMNH 1877.5.21.142, **A** habit **B** various calthrops shapes **C** toxas, including several malformations **D** various sanidaster shapes.

Figs 2A–D 3A–E 5a-e Table 1

Halina bucklandi Bowerbank 1858: 288.
Hymeniacidon bucklandi ; Bowerbank 1866: 226; 1874: pl. 38 figs 9–12.
Dercitus bucklandi ; Gray 1867: 542; Topsent 1895: 528; Ackers et al. 1992 (2007): 52; Van Soest et al. 2008 (CD-ROM).
Pachastrella bucklandi ; Schmidt 1870: 76.
Dercitus niger Carter 1871: 3, pl. IV fig.1.
Battersbyia bucklandi ; Bowerbank 1874: 346, pl. 92 fig. 8; Bowerbank and Norman 1882: 93.

####### Material examined.

Schizoholotype (2 slides, one with dissociated spicules, the other with section at right angles to surface): NHM collection, Bwbk. 542 (labeled as Battersbyia bucklandi, Hymeniacidon bucklandi), part of holotype BMNH 1877.5.21.142 (wet, fragment examined); locality presumed to be Abbey Bay, near Torquay, Devon, England (but this is not indicated on the labels).

**Figure 3.**
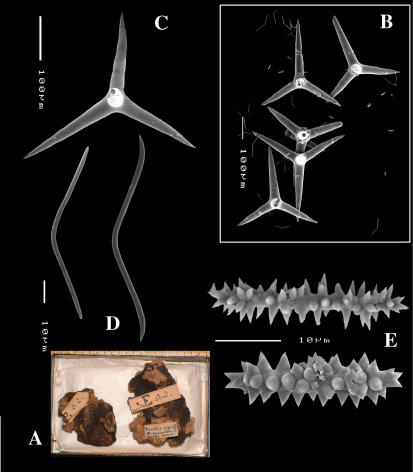
Dercitus (Dercitus) niger (= j.syn. of Dercitus (Dercitus) bucklandi), type specimens, BMNH 1895.4.27.1–6, **A** habit **B** overview of spicule complement **C** calthrops **D** toxas **E** sanidasters.

Holotype of Dercitus niger Carter, 1871 (dry) BMNH 1895.4.27.1–6 (Fig. 3) ; dry schizotype ZMB 3046, (including 17 slides); locality Straight Point, Budleigh–Salterton, South Devon.

####### Description

(amalgamated from various descriptions of material from the British Isles and NW France). Cushion-shaped to massive-lobose (Figs 2A, 3A), filling crevices in vertical rock faces. Size frequently over 5cm2. Black to dark grey-brown externally. Surface smooth but often has ridges looking like stretch-marks. The surface is usually concave. Apertures (oscules?) are flush with the surface, variable in size and usually collected into groups towards the centre of the sponge. Consistency moderately firm but compressible and spongy when in situ, liver-like in preserved condition.

Skeleton: Main skeleton of confused, randomly arranged calthrops, a layer of sanidasters and many toxas occur near the surface, but these also occur in the choanosome. There are many large pigment cells (?) with black inclusions evident in sections.

Spicules: Calthrops, toxiform microscleres, sanidasters.

Calthrops (Figs 2B, 3B–C), often with axial canals seen clearly, occasional bifid cladi or with angulated curve, rarely with 5 cladi, size of cladi: 80–*218.9*–305 × 12–*28.1*–48 µm, cladomes 120–*311.3*–480 µm.

Smooth toxiform microscleres (Figs 2C, 3D, 5a–e), often slightly swollen near the apices, occasionally with irregular side branches (Fig. 2C) : 51–86.8–111 × 1–2 µm.

Sanidasters (Figs 2D, 3E) relatively robust with strong spines in ‘mature’ condition : 22–*25.7*–31 × 0.5–*5.2*–7 µm (spines included in width).

**Table 1.** Spicule data reported for specimens of Dercitus bucklandi, upper part: Dercitus (Dercitus) bucklandi bucklandi, lower part Dercitus (Dercitus) bucklandi lusitanicus ssp. n. (*denotes computed lengths, see text).

Author	calthrops cladi	cladome	sanidasters	toxas	locality
Bowerbank 1858, 1866, 1874 BMNH 1877.5.21.142	81–318 × 24–41	120–480	22–31 × 0.5–7	64–111 × 1–2	Devon Guernsey
Carter 1871 BMNH 1895.4.27.1–6 & ZMB 3046	108–356 × 17–37	142–472	21–31	51–103	Devon, UK
Topsent 1895	310–320 × 38–45	465–480*	21–27 × 6–8	75–90 × 2–3	Bretagne
Ferrer 1918	95–210 × 30	142–310*	not reported	not reported	Asturias
Xavier and van Soest 2007	58–119 ×5–16	126–186	17–22 × 0.5–3	37–58 × 0–1	Gorringe
Berlengas unpubl.	75–165 × 7–27	110–240	16–24 × 1.5–2	42–69 × 0.5–1.5	Lagosteira
Portuguese main coast unpublished	118–135 × 12–22	175–200	19–21 × 2	48–51 × 0.5–1.5	Arabida

####### Habitat.

In crevices in vertical rock faces in clean water. Particularly common on limestone substrata. From the extreme low water mark to a few meters subtidally in rock pools and caves.

####### Distribution.

British Isles (SW coast of England and N and W coast of Ireland), and France (Roscoff, Iles de Glénan). There is a record from the northern Gulf of Mexico (Teerling 1975), but this has not been verified and is unlikely to be correct.

####### Etymology.

Named after Mrs. Buckland of Guernsey, who collected one of the type specimens (in fact the species should probably have been named “bucklandae” since the named person is female).

####### Remarks.

Bowerbank’s type specimen (a fragment, BMNH 1877.5.21.142, Fig. 2A) and slide (Bwbk. 542 ) and two of Carter’s (1871) types of Dercitus niger (BMNH 1895.4.27.1–6, Fig. 3A, and ZMB 3046) were reexamined (see also Table 1). Bowerbank’s material (Fig. 2) had calthrops with cladi 81–318 × 12–48 µm (cladome 120–480 µm), toxas 64–111 × 1–2 µm, and sanidasters 22–31 × 0.5–7. In the type material, the toxas showed some peculiar straight or curled side branches usually in the mid section (see below).

Carter’s type specimens (Fig. 3) possessed spicule sizes closely similar to those of Bowerbank’s type: calthrops 108–356 × 17–37 µm (cladome up to 472 µm), toxas 51–103 × 1.5–3 µm, sanidasters 21–31 × 3–7 µm. However, there was a curious discrepancy in shapes of calthrops and the sanidasters between the BMNH and ZMB Carter type specimens. Presumably this conveys a large variability among specimens from the same locality.

Topsent (1895) gives the following measurements of specimens from the W coast of France: calthrops with cladi 310–320 × 38–45 µm (thicker than the types), toxas 75–90 (similar to the types), sanidasters 21–27 µm (similar to the types).

In view of some of the discrepancies we considered it worthwhile to investigate whether spicular differences of specimens within the known range of the species would yield a pattern that could explain some of the differences. We compared spicular data from the northern samples cited above with those of samples from areas in the southern part of the range, from the coasts and offshore localities of Portugal obtained from Dr Joana Xavier and examined spicule sizes and shapes. This led us to the conclusion that there are consistent differences between samples from these areas which should be recognized at the subspecies level.

###### Dercitus (Dercitus) bucklandi subsp. lusitanicus ssp.n.

urn:lsid:zoobank.org:act:9152BD64-067E-4F21-AF03-6B84E671E2A6

**Figure 4.**
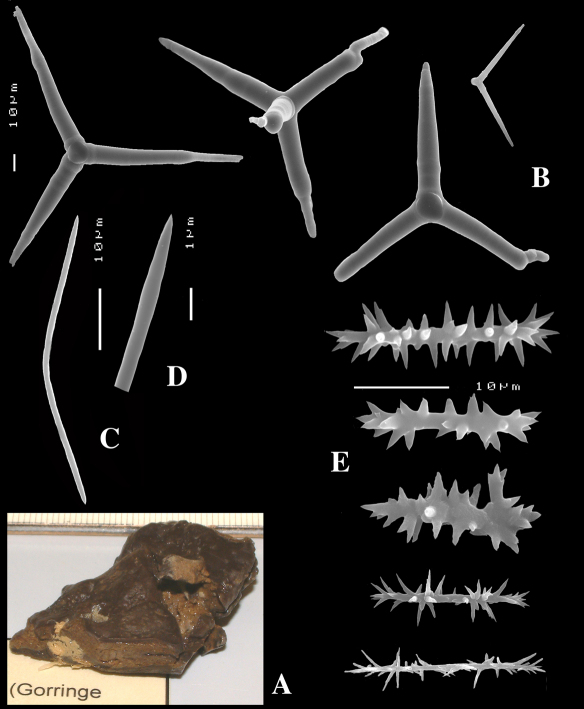
Dercitus (Dercitus) bucklandi lusitanicus ssp. n., holotype ZMA Por. 21810 from Gorringe Bank, **A** habit **B** various shapes and sizes of calthrops **C** toxa **D** detail of toxa **E** various shapes of sanidasters.

Figs 4A–E 5f-j Table 1

Dercitus bucklandi ; Ferrer-Hernandez 1918: 17; Rodriguez Babio and Gondar 1978: 34; Acuña et al. 1984 (in Solórzano 1991: 21); Templado et al. 1986: 96 (table); Xavier and Van Soest 2007: 1646.

####### Material examined.

Holotype ZMA Por. 21810, Portugal, Gorringe Bank, Gettysburg Peak, 31–38 m, coll. J. Xavier, 2006 (cf. Xavier and Van Soest 2007, Figs 4A–E).

Additional specimens. Xavier collection, field nr. B05.09.36, Portugal, Berlengas Archipelago, Lagosteira, 7 m, 16–IX–2005, coll. J.R.B.T. Xavier.

Xavier collection, field nr. B05.09.59, Portugal, Berlengas Archipelago, Lagosteira, 6–7 m, 16–IX–2005, coll. J. Cristobo.

Xavier collection, Portugal, field nr. B05.09.98, Berlengas Archipelago, Lagosteira, 6 m, 18–IX–2005, coll. J.R.B.T. Xavier.

Xavier collection, field nr. B05.09.267, Portugal, Berlengas Archipelago, Gruta do Carreiro Maldito, 6–8 m, IX–2005, coll. A. Cunha.

Xavier collection, Portugal, field nr. A03/73, Arabida, Ponta da Passagem, 7 m, 16–VII–2003, coll. J.R.B.T. Xavier.

####### Description

(Fig. 4A). Alive blackish outside and greyish inside (in alcohol chocolate brown throughout). Shape massively encrusting, flat with smooth surface, no apparent oscules; inhalant openings in sieve plates. Consistency cheesy, slightly compressible. A representative size of preserved specimens is 5×4×0.7 cm.

Skeleton: a layer of microscleres overlying a loose mass of calthrops megascleres. Spicular density appears lower than in the specimens from the British Isles.

Spicules: calthrops, toxas, sanidasters.

Calthrops (Fig. 4B), relatively uniform in size, cladi of irregular outline with endings irregularly stair-stepped and/or malformed, occasionally one or more cladi lacking; 58–*108.3*–165 × 5–*14.6*–27 µm, cladome 110–*159.3*–240 µm.

Toxa-like microscleres (Figs 4C–D), symmetrical, smooth, but slightly undulate / polytylote, with a shallow curve and evenly pointed; 37–*51.3*–69× 0.5–*0.7*–1.5 µm.

Sanidasters (Fig. 4E), rather uniform in size and shape, spines relatively long and thin, occasionally with a somewhat irregular shape; 16–*19.3*–24 × 0.5–*2.4*–6 µm (including spines).

**Figure 5.**
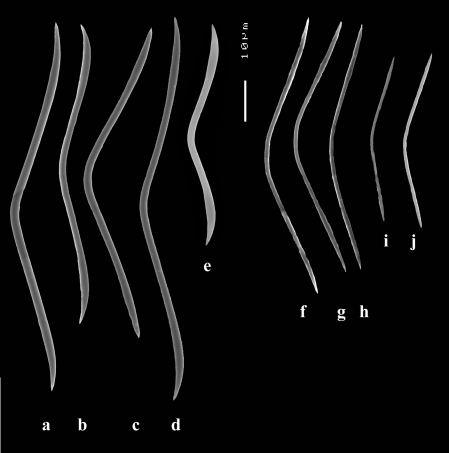
Dercitus (Dercitus) toxa microscleres, a–e. Dercitus (Dercitus) bucklandi bucklandi from Celtic Seas, f–j. Dercitus (Dercitus) bucklandi lusitanicus ssp. n. from Lusitanian waters.

####### Habitat.

Encrusting, typically bridging crevices and gaps in the substrate, sublittoral down to 6–38 m.

####### Distribution.

Portuguese main coast, Berlengas Islands, Gorringe Seamount. Furthermore, there are reports from the north coast of Spain (Ferrer-Hernandez, 1918; Babio & Gondar, 1978 (not seen); Acuña et al. 1984 (not seen). There is also a record from the Alboran Sea (Western Mediterranean) by Templado et al. 1986.

####### Remarks.

The spicule measurements of the present specimens are generally significantly smaller than those provided by most previous authors (Table 1), which is especially clear in the sizes of the toxiform microscleres: 51–111 × 1.5–3 µm in the subspecies *bucklandi*, 42–69 × 0.5–1.5 µm in the samples of the new subspecies, compared in Fig. 5: with *bucklandi bucklandi* toxas in Figs 5a–e and *bucklandi lusitanicus* ssp.n. toxas in Figs 5f–j. Possibly, the southern samples may be distinguished at the species level from the northern samples, but in view of the fact that there is an overall strong similarity with Dercitus bucklandi, we prefer to recognize only subspecies.

The toxa–like microscleres of Dercitus bucklandi s.l. are unique in the Astrophorida. They have been called ‘toxa’ because of the similarity in shape to similar microscleres in microcionid and mycalid Poecilosclerida. However, the resemblance is probably superficial and it is unlikely that these are homologous spicule types. There are subtle differences mostly only clearly visible in SEM images, such as the tendency to become apically swollen, and an overall faint ‘polytyly’ (Fig. 4D), and – at least in the Bowerbank type material of the nominal subspecies – the not infrequent presence of peculiar side branches or single long spines near the curved part in the middle. Possibly, this indicates that the microsclere derives from an end to end fusion of two incipient smooth microxeas – a more likely assumption than Topsent’s (1895) suggestion that the toxas are modified asters –, but firm proof for this hypothesis is wanting. Smooth microxeas are relatively common in several ancorinid genera.

##### Subgenus Stoeba

Dendy, 1905

###### Definition:

Dercitus with a single microsclere category in the form of irregular sanidasters.

###### Type species:

Samus simplex Carter, 1880.

###### Key to taxa of subgenus Dercitus (Stoeba).

**Table:** 

1	Megascleres exclusively calthrops, no proper dichocalthrops (but bifid cladi may be present in the calthrops)	2
–	Megascleres exclusively dichocalthrops, no proper calthrops	8
–	Megascleres including both dichocalthrops and calthrops	10
2	Longest cladi of calthrops less than 200 µm	3
–	Longest cladi over 200 µm	5
3	Longest cladi of calthrops less than 100 µm	4
–	Longest cladi of calthrops up to 200 µm	Dercitus (Stoeba) sp. Bonaire
4	Calthrops exclusively three-claded, may be absent; sanidasters up to 20 µm	Dercitus (Stoeba) xanthus
–	Calthrops both normal and three-claded, always present; sanidasters 8–12 µm	Dercitus (Stoeba) syrmatitus
5	Longest cladi of calthrops over 250 µm (maybe up to 400 µm)	Dercitus (Stoeba) senegalensis sp. n.
–	Longest cladi of calthrops less than 250 µm	6
6	Sanidasters up to 25 µm long	Dercitus (Stoeba) sp. aff. *plicatus* Malaysia
–	Sanidasters only 10–15 µm long	7
7	Live colour red (dark in preservation)	Dercitus (Stoeba) latex
–	Live colour yellow (white in preservation)	Dercitus (Stoeba) sp. Madagascar
8	Cladome only up to 230 µm in diameter	Dercitus (Stoeba) occultus
–	Cladome up to 450 or more µm in diameter	9
9	Insinuating in calcareous algae, colour brownish	Dercitus (Stoeba) simplex
–	Encrusting, white	Dercitus (Stoeba) verdensis sp. n.
10	Calthrops very large, cladi up to 648 µm	Dercitus (Stoeba) reptans
–	Cladi of calthrops less than 250 µm	11
11	Calthrops with one cladus distinctly longer	Dercitus (Stoeba) bahamensis sp. n.
–	Cladi may be unequal in length but none are distinctly longer	12
12	Dichocalthrops distinctly smaller than calthrops	13
–	Dichocalthrops similar in cladome size as calthrops	14
13	Sanidasters with low short spines	Dercitus (Stoeba) lesinensis
–	Sanidasters profusedly spined	Dercitus (Stoeba) plicatus s.s.
14	Largest dichocalthrops cladomes over 300 µm	Dercitus (Stoeba) fijiensis sp. n.
–	Largest dichocalthrops cladomes less than 300 µm	15
15	Sanidasters fusiform, pointed	16
–	Sanidasters rhabd-like, blunt	17
16	Colour pink, thickness of cladi of megascleres 10 µm	Dercitus (Stoeba) pauper
–	Colour grey, thickness of cladi of megascleres up 27 µm	Dercitus (Stoeba) extensus
17	Oxeas present	Dercitus (Stoeba) dissimilis
–	No oxeas	Dercitus (Stoeba) sp. aff. *plicatus* Maldives

###### Dercitus (Stoeba) simplex

(Carter, 1880)

Figs 6A–B

**Figure 6.**
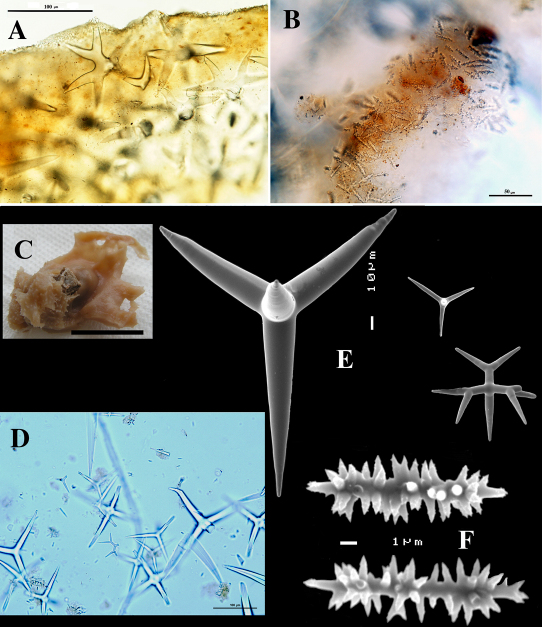
Dercitus (Stoeba) microphotos, A–B. Dercitus (Stoeba) simplex, BMNH 1931.1.1.31a, slide of specimen from Invisible Bank, Andaman Islands, **A** overview of dichocalthrops (scale bar 100 µm) **B** sanidasters (scale bar 50 µm) **C–F** Dercitus (Stoeba) plicatus, holoype MNHN DT 3635, with schizotype BMNH 1868.3.2.1. (slide only), from Algiers 1868, **C** photo of holotype, made by P. Cárdenas (scale bar 1 cm) **D** overview of spicules in BMNH type slide (scale bar 100 µm) **E** SEM photo of megascleres from holotype **F** sanidasters from holotype.

Samus simplex Carter 1880: 60, pl. V fig. 26.
Stoeba simplex ; Sollas 1888: 102; Maldonado 2002: 155, fig. 12A–B.
Dercitus simplex ; Thiele 1900: 20, pl. II fig. 1.
Stoeba plicata  var. *simplex*; Annandale 1915: 458.
Dercitus plicatus var. simplex ; Burton and Rao 1932: 309.
Halina plicata ; Thomas 1972: 353, pl. II figs 6A–B.

####### Material examined.

BMNH 1931.1.1.31a, Invisible Bank, Andaman Islands, slide only of which it is uncertain whether it represents the present species. Type: Indian Museum Calcutta ? (not seen).

####### Description

(from Carter and Sollas). “Sponge excavating. Spicules: Megasclere dichotriaene, rhabdome 0.210 × 0.042 mm. Microsclere rod-like with numerous small spines 0.0127 mm in length. Habitat: Gulf of Manaar”.

####### Remarks.

The exact properties and variation of this species have not been established so far, so we can only provide a discussion pending a proper revision. The type material has not been reexamined and its precise whereabouts are uncertain.

Annandale (1915) described specimens from the Mergui Archipelago (now Myanmar) as similarly ‘excavating’ (they are insinuating dead corals and filling spaces presumably made by clionaid sponges) and having dichocalthrops, and a single calthrops, as megascleres (sizes not given); sanidasters 16.2 µm.

Thomas (1972) described ‘coral boring’ material from the Gulf of Manaar, insinuating, with dichocalthrops having protocladi 33×2, deuterocladi 79×16, rhabdome 63×16 and cladome 210 µm. The rhabdome is clearly shorter than in the other described specimens.

In contrast to previous descriptions, Thiele (1900) recorded this species from Ternate (Indonesia, Halmahera) as forming a black encrustation likened to Dercitus (Dercitus) bucklandi. Spicule dimensions provided were: rhabdome of dichocalthrops 150 × 25 µm, cladome also similar in size, sanidasters 9 µm, provided with ‘numerous clear spines’. Thiele also mentions the presence of large (12–35 µm) pigment cells.

Maldonado (2002) gave a description of an Indian specimen from the Andaman Islands, supposedly of this species, but not the Gulf of Manaar type specimen itself. It is presumed that he described the specimen mentioned in Burton and Rao (1932). We borrowed the same slide from the NHM collection (BMNH 31.1.1.31a). It is possible that this specimen which according to Burton and Rao (l.c.) in external form resembled Dendy’s (1905) Dercitus (Stoeba) extensus more than Dercitus (Stoeba) simplex may indeed have been of another species as there is a big difference between the original description of Carter and the Burton and Rao slide in the size of the microrhabds: 12.7 µm in the type, 18–28 × 3–7 µm in the Andaman specimen (Fig. 6B). The rhabd of the dichocalthrops of the type is given as 210 × 42 µm whereas Maldonado gives 40–225 × 60–75 µm in the Andaman specimen (remeasured by us 75–*251.5*–317 × 12–*39.8*–52 µm) (Fig. 6A). Maldonado additionally gives protocladi as 30–50 × 12–60 µm (remeasured by us: 53–*84.3*–117 × 14–*39.7*–51) and deuterocladi as up to 150 × 35–40 µm (remeasured 15–*105.0*–157 × 8–*29.4*–38 µm). Cladomes 128–*386.8*–492 µm. Small dichocalthrops were rare in the slide.

If all these specimens are members of a single species, then it occurs on both sides of the Gulf of Bengal as well as on the islands in the middle of it and to the east into Indonesia. However, specimens need to be reexamined.

###### Dercitus (Stoeba) plicatus

(Schmidt, 1868)

Figs 6C–F

**Figure 7.**
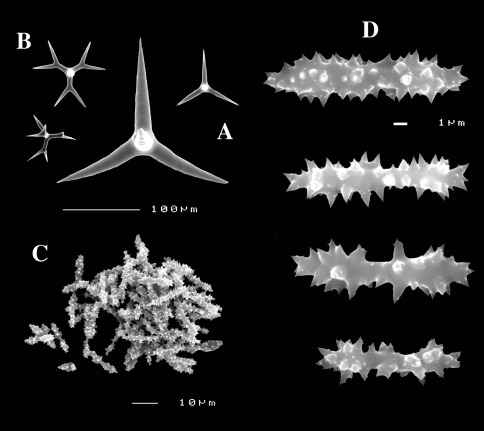
Dercitus (Stoeba) lesinensis, syntype ZMB 2409, dredged near Lesina, Croatia, **A** various calthrops **B** various dichocalthrops **C** mass of sanidasters **D** various sanidasters.

**Figure 8.**
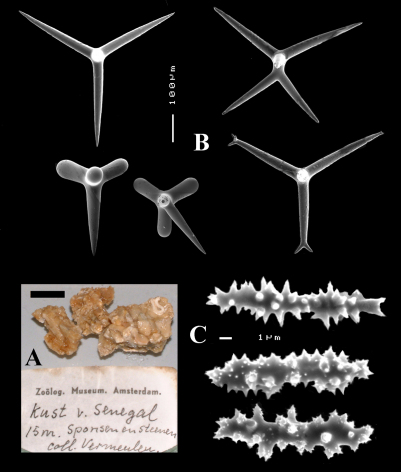
Dercitus (Stoeba) senegalensis sp. n., holotype ZMA Por. 06721, Senegal, **A** habit (scale bar 1 cm) **B** calthrops including variations in shape and number of cladi **C** various sanidasters.

**Figure 9.**
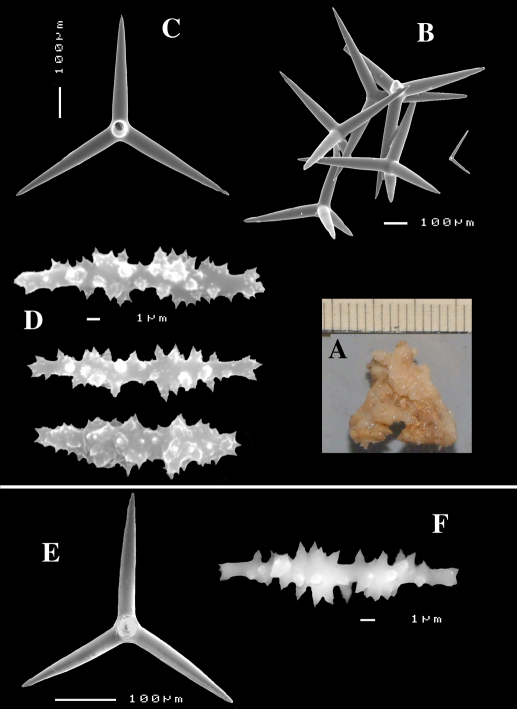
Dercitus (Stoeba) senegalensis sp. n., **A–D** Paratype ZMA Por. 06721, Mauritania, off Banc d’Arguin, **A** habit, **B** overview of megascleres **C** calthrops **D** various sanidasters **E–F** Possible additional member of Dercitus (Stoeba) senegalensis sp. n., ZMA Por. 21697, Aegean Sea **E** calthrops **F** sanidaster.

**Figure 10.**
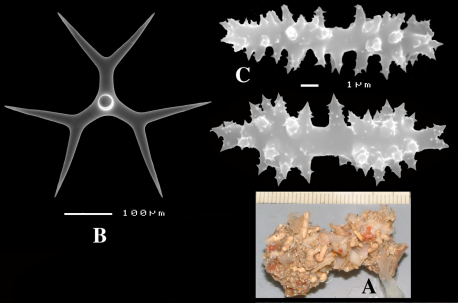
Dercitus (Stoeba) verdensis sp. n., holotype ZMA Por. 07521b, from Cape Verde Islands, **A** habit consolidating calcareous substratum **B** dichocalthrops **C** sanidasters.

**Figure 11.**
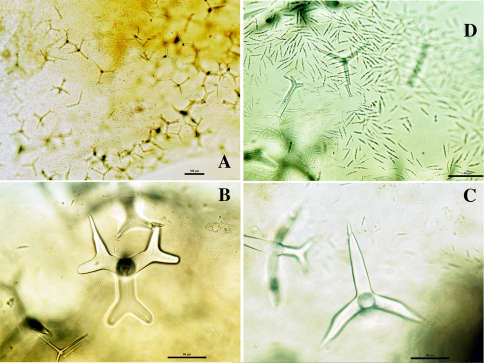
Dercitus (Stoeba) extensus, slides from type BMNH 1907.2.1.5a, Gulf of Mannaar, Sri Lanka, **A** overview of peripheral skeleton (scale bar 100 µm) **B** dichocalthrops (scale bar 50 µm) **C** calthrops (scale bar 50 µm) **D** surface view of sanidasters (scale bar 50 µm).

Corticium plicatum Schmidt 1868: 2, pl. III fig. 11.
Calcabrina plicata ; Sollas 1888: 281.
Dercitus plicata ; Lendenfeld 1894: 17, pl. II fig. 10, l. III fig. 43.
Dercitus plicatus ; Topsent 1895: 531, pl. XXII figs 6–10; Babiç 1922: 286, fig. V-1; Lévi and Vacelet 1958: 231, Figs 10–11; Pulitzer-Finali 1972: 345; Pulitzer-Finali 1983: 467; Templado et al. 1986: 96 (table); Pansini 1987: 157; Van Soest 1993: 210 (table).

####### Material examined.

Holotype MNHN DT 3635, labeled “Corticium plicatum Schmidt 1868 no. 96” (there is also a slide – not examined - with Schmidt’s handwriting, bearing the date 1868); schizotype BMNH 1868.3.2.1. (slide), labeled “Corticium plicatum Schmidt, 1868, Calcabrina plicata, Algiers 1868”.

Additional specimen ZMA Por. 15101a, Banyuls, ‘coralligène’, 42.4833°N; 3.138°E, 10 m, coll. S. Groot, 10 August 1981.

####### Description.

The wet holotype (Fig. 6C) consists of two pieces, one small brownish mass of 2×2 cm, the actual specimen, and a larger limestone mass covered with bryozoans which does not appear to contain any additional sponge material but is presumed to be part of the substratum on which the sponge grew. The holotype is slightly fleshy, and in cross section consists of an outer layer of sanidasters overlying a dense confused mass of calthrops. Later descriptions (e.g. Topsent 1895) diagnose this species as encrusting and insinuating between stones and calcareous algae. Colour white, interior yellowish. Consistency firm, collagenous. Surface smooth with single oscules elevated into small conical papillae. Numerous large cells with inclusions (70 µm).

Spiculation of the holotype (Figs 6E-F, Table 2): Calthrops, dichocalthrops, sanidasters.

**Table 2.** Spicule data reported for specimens of Dercitus (Stoeba) plicatus (*denotes computed lengths, see text).

a. Specimens from the Northeastern Atlantic-Mediterranean area				
Author	calthrops	dichocalthrops	sanidaster	locality
Schmidt 1868 holotype MNHN D.T. 3636	41–188 × 3–29 cladome 57–252	prot. 20–28 deut. 15–36 rhabd 28–57 cladome 67–105	11–19 × 1–2	Algeria
Schmidt 1868 Schizotype BMNH 1868.3.2.1	36–183 × 4–28 cladome 62–258	prot. 14–21 deut. 21–33 rhabd 36–45 cladome 73–93	11–18 × 1–2.5	Algeria
Sollas 1888	60 cladome 90*	present (rare)	8.3	Algeria
Lendenfeld 1894	60–80 × 8 cladome 90–120*	present (small)	6–7 × 1.5	Adriatic
Topsent 1895	170–200x 25–30 cladome 255–300*	present	12–15 × 2–3	Banyuls
Babiç 1922	50–238x 18–35 cladome 75–357*	prot.15–18 deut. 22–100 rhabd 50–120 cladm 72–218*	10–16 × 2–3	Adriatic
Lévi and Vacelet 1958	60–250 × 30 cladome 90–375*	prot.25–30 deut. 20–70 rhabd 40–80 cladome 80–190*	12–13	Portugal (Gettysburg)
Pulitzer-Finali 1972	40–60 (rare) cladome 60–90*	prot.not given deut.not given rhabd not given cladm 60–210	10–20	Italy
Pulitzer-Finali 1983	40–200 cladome 60–300*	prot.20–40 deut.25–50 rhabd not given cladm 80–180*	12–16×2.5	Italy
Boury-Esnault and Lopes 1985	30–206×3–23 cladome 45–309*	cladi 44–65 rhabd 75–80 cladm 80–125*	11–18 × 0.7–1.5	Azores
ZMA 15101 unpublished	56–195 × 7–20 cladome 92–238	proto 24–31 deut. 15–21 rhabd. 35–40 cladome 78–98	16–22 × 1.5–2	Banyuls
b. Specimens described as different species suspected to be D. plicatus.				
Dercitus lesinensis (includes oxeas)	92–165 × 14–31 cladome 180–210	proto 16–18 deut. 15–32 rhabd 45–50 cladome 60–74	11–18 × 2–3	Adriatic
Nethea dissimilis (includes oxeas)	45–175 × 5–21 cladome 67–263*	cladome 77–102	8–15 × 0.7–4	Naples
c. Specimens originally identfied as D. plicatus here assigned to new species.				
Dercitus senegalensis sp. n. Van Soest, 1993 (list) ZMA 21350 (holotype)	92–426 × 8–55 cladome 126–618	not present	11–17 × 1–4	Senegal
Van Soest 1993 (list) ZMA 06721 (paratype)	36–480 × 6–60 cladome 61–810	not present	12–21 × 1.5–3	Mauritania
Lévi 1952	250 × 30 µm	not present	10–15 × 2	Senegal
ZMA 21697 so far unpublished	141–295 ×11–48 cladome 228–475	not present	13–21 × 1–2	Aegean Sea
Dercitus verdensis sp. n. Van Soest,1993 (list) ZMA 07521b	not present	prot. 42–56 deut. 23–204 rhabd. 71–252 cladome 132–475	11–16 × 1–2	Cape Verde Is.
Dercitus fijiensis sp. n. ZMUC unnumbered	96–258 × 198–37 clad.186–420	prot. 19–30 deut. 55–192 rhabd. 58–132 clad. 129–361	15–21 × 3–4	Fiji
d. Specimens from the Indo-West Pacific suspected to belong to undescribed species				
Sollas 1902 BMNH? not found	100–240 × 15–27 cladome 150–360*	not present	12–25 × 2–3	Malaysia
MSNG MD6,MD2 Calcinai et al. 2000	70–90 cladome rare	prot.12–25 105–135*deut. 20–82 rhabd. 41–102 cladm.60–200*	8–13 × 2–3	Maldives

Calthrops (Fig. 6E) are dominating the megasclere complement, in a wide size range, but not divisible in size classes, most are regular four-claded equal-length spicules, a few three-claded occur and occasionally cladi are a bit crooked, cladi 41–*101.0*–188 × 3–*14.7*–29 µm, cladomes 57–*154.4*–252 µm.

Dichocalthrops (Fig. 6E), relatively rare, only a dozen were encountered in the spicule slide made from the holotype, invariably smaller than the biggest calthrops; infrequent incipient dichocalthrops, were encountered, with only two or one of the cladi bifid. Size of protocladi 20–*22.4*–28 × 4–*5.6*–8 µm, deuterocladi 15–*28.0*–36 × 2–*3.6*–6 µm, rhabds 28–*45.6*–57 × 3–*5.6*–9 µm, and cladomes 67–*86.8*–105 µm.

Sanidasters (Fig. 6F) are slightly fusiform and profusedly spined, relatively uniform in shape and size, 11–*14.9*–19 × 1–*1.65*–2 µm.

Additionally, we observed some oxeas of uniform size, approximately 150 × 3–4 µm, assumed to be foreign to the sponge.

Description of the BMNH Schmidt’s type slide (Fig. 6D, Table 2). The spicules (calthrops, dichocalthrops and sanidasters) are dissociated, but even in that condition it can be concluded that the slide is almost certainly taken from the holotype as the frequencies of occurrence and the sizes of the spicules are very similar to those of the holotype. Calthrops dominate the megascleres (Fig. 6C); most are regular, but occasionally some are three-claded or rarely two-claded. Endings of the cladi may occasionally be abruptly bent, bifid, indicating incipient dichocalthrops. They occur in a large size range, cladi 36–183 × 4–28 µm, cladomes 62–258 µm, almost identical in range to the spicules measured from the holotype. Dichocalthrops rare, as the slide contained only four measurable dichocalthrops, protocladi 15–21 × 3.5–10 µm, deuterocladi 21–33 × 3–8 µm, rhabds 73–95 µm, cladomes 36–45 µm. Sanidasters 11–18 × 1–2.5 µm.

The only non-type specimen available to us, ZMA Por. 15101 from Banyuls has spiculation closely similar to the type material (Table 2). Only the sanidasters appear on average slightly longer and more robust.

####### Habitat.

Encrusting and insinuating in crevices, large depth range down to 100 m.

####### Distribution.

Originally reported from Algeria. Elsewhere reported with certainty from Banyuls, Naples and the Adriatic. Possibly some of the records from the adjacent North Atlantic (Lévi and Vacelet 1958; Boury-Esnault and Lopes 1985) also refer to the present species, but see below.

####### Remarks.

Spicule dimensions of reported specimens are presented in Table 2. Sollas (1888), Lendenfeld (1894) and Pulitzer-Finali (1972) give unusually small calthrops (cladi 60–80 µm). Sollas (1888) and Lendenfeld (1894) additionally give very small sanidasters (8.3 and 6–7 µm). Topsent (1895) gives spicule data for this species as follows: calthrops with cladi 170–200 µm; dichocalthrops with similar sized cladi, with rabd thickness 25–30 µm; sanidasters 12–15 × 2–3 µm. Pulitzer-Finali (1983) reports this species from various Western Mediterranean localities and depths 1–100 m. Colours reported were white, pink, violet and brown. Both calthrops (cladi 40–200 µm) and dichocalthrops (cladome 60–210 µm) were present in variable quantities. Sanidasters varied between 10 and 20 µm. Pansini (1987) reports white specimens from the Alboran Sea lacking dichotriaenes, but provided no further data. It is possible this record concerns Dercitus (Stoeba) senegalensis sp. n. (see below).

Dercitus (Stoeba) plicatus is apparently quite variable and this may have led to widespread reports of the species from various East Atlantic localities, but also from Malaysia (Sollas 1902), Gulf of Mannar (Thomas 1970), Maldives (Calcinai et al. 2000), and Fiji (Tendal 1969).

We believe alleged records of this species outside the Mediterranean (Table 2) need to be reviewed critically on the basis of reexamination of the specimens. We have done so for material available to us from West African and Fijian localities (see below). In these specimens we found important differences with Mediterranean Dercitus (Stoeba) plicatus, which led us to assign them to three new species. It is likely that further records from e.g. Indo-West Pacific localities which we could not verify are part of a complex of Dercitus plicatus-like sister species distributed over most of the warmer parts of the oceans.

Several species of Dercitus (Stoeba) were synonymized with the present species by various authors, including the type species Dercitus (Stoeba) simplex. In most cases these synonymies are not accepted by us.

###### Dercitus (Stoeba) lesinensis

(Lendenfeld, 1894)

Figs 7A–D

Pachastrella lesinensis Lendenfeld 1894: 18, pl. II fig. 18, pl. III fig. 44, pl. IV figs 67–68.
Dercitancorina lesinensis ; Topsent 1902: 15.
Stoeba lesinensis ; Maldonado 2002: 156.

####### Material examined.

Syntype fragment ZMB 2409, dredged near Lesina, Croatia.

####### Description.

Encrusting and insinuating among stones and coralline algae, with oscular elevations. The fragment examined by us was a thin rounded papilla-like extension (probably from the larger of the two type specimens described by Lendenfeld). Consistency slightly rubbery. Colour (alcohol) orange.

Skeleton: at the surface consisting of a dense crust of microscleres, subdermally the skeleton consists of a mass of calthrops, embedded in a fibrous-organic groundmass.

Spicules: calthrops, dichocalthrops and sanidasters, no complete oxeas are found in the fragment examined, but a few broken monaxones were present.

Calthrops (Fig. 7A), with occasional bifid cladus endings, cladi 92–*130.9*–165 × 14–*21.3*–31 µm (Lendenfeld: up to 160 × 40 µm), cladomes 180–210 µm.

Dichocalthrops (Fig. 7B), rare, small, mentioned by Lendenfeld, but no measurements given. Our preparations contained a few, with protocladi 16–18 × 3–8 µm, deuterocladi 15–32 × 3–6 mm, cladome diameters 70–74 µm, rhabdi 40–47 µm.

Sanidasters (Figs 7C–D), with short low spination, size 11–*15.2*–18 × 2–*2.45*–3 µm (Lendenfeld: 12–15 × 1.6 µm).

####### Habitat.

No data.

####### Distribution.

Adriatic. So far there is only a single record of this species.

####### Remarks.

A major difference with the original description is the lack of giant oxeas (4000 × 70 µm), some centrotylote, reported and pictured by Lendenfeld (1894); also tylostyles and strongyles were reported. The fragment of the type specimen received from ZMB did not contain any such spicules, except for some broken fragments. For Maldonado (2002) the alleged presence of oxeas in a species otherwise considered by him a typical Stoeba was the reason to accept species with structural monaxone megascleres within the definition of Stoeba. We maintain that such spicules, if they would be proper, are auxiliary, not structural.

This species is similar in most respects to Dercitus (Stoeba) plicatus, but the orange colour has not been reported for that species (although Pulitzer-Finali (1983) mentions brown specimens of Dercitus (Stoeba) plicatus). Based on our observations of the sanidasters, it is possible that these are slightly different from Dercitus (Stoeba) plicatus in spination, with the latter having more profused spines. In view of this, we are forced to retain Dercitus (Stoeba) lesinensis as a valid species for the time being.

###### Dercitus (Stoeba) dissimilis

(Sarà, 1959)

Nethea dissimilis Sarà 1959: 3, fig. 1.
Stoeba dissimilis ; Maldonado 2002: 156.

####### Material examined.

None (no response from an online form request to the Stazione Zoologica, Naples).

####### Description

(from Sarà 1959). Numerous specimens (but only one was preserved, the holotype GG907, present whereabouts unknown) in a cave, depth 0–7 m, 30 m from the entrance. Whitish encrustations filling interstices between oysters and barnacles. Individual size 1–6 cm2, total size of all specimens 43 cm2.

Skeleton: no data.

Spicules: calthrops, dichocalthrops, sanidasters, oxeas (but see discussion).

Calthrops with the fourth cladus often shorter, occasionally triactines, cladi 45–175 × 5–21 µm.

Dichocalthrops, cladome 77–102 µm, thickness of cladi 3.5–6 µm, no individual proto-, deuteroclad or rhabd measurements provided.

Sanidasters, amphiaster-like with spines concentrated on both sides, length 8–15 µm, thickness 3–4 µm (with spines) 0.7–1.5 µm (without spines).

Oxeas, extreme size variation, 65–930 × 1.8–21 µm, no indication what their structural position is within the sponge.

**Habitat**. In shallow-water caves.

**Distribution**. Naples, Italy.

**Remarks**. Because no material has been examined and previous authors assigned this to Stoeba we retain this as a valid species of Dercitus (Stoeba) for the time being on account of the reported oxeas. However, apart from the oxeas the description perfectly fits that of Dercitus (Stoeba) plicatus (Schmidt, 1862) (see above). Especially the white colour, the Mediterranean occurrence and the combined presence of dichocalthrops and calthrops are telltale signs that they could be conspecific.

###### Dercitus (Stoeba) senegalensis sp. n.

urn:lsid:zoobank.org:act:1243255F-E761-4220-99AA-1CA8C9826132

Figs 8A–C 9A–D

Dercitus plicatus ; Lévi 1952: 42, fig. 5; Van Soest 1993: Table 2, pars (not: Dercitus plicatus sensu Schmidt 1868).

####### Material examined.

Holotype ZMA Por. 21530, off the coast of Senegal, coll. F.P. Vermeulen, 1906, fish trawl.

Paratype ZMA 06721, Mauritania, off Banc d’Arguin, 19.0667°N; 16.4167°W, dredged at 12–18 m, coll. R.W.M. Van Soest, R.V.’Tyro’ Mauritania II Expedition stat. 049, 11 June 1988.

Additional (non-type) material ZMA Por. 21697, Aegean Sea, fragment of specimen nr. 101 of E. Voultsiadou’s collections.

####### Description

(Figs 8A, 9A). Sponge encrusting and agglutinating calcareous fragments, colour dirty white. Lateral size approx. 5×3 cm. The holotype now consist of three fragmented pieces, the paratype is 1.5×1.5 cm. Consistency crumbly.

Skeleton: a confused mass of calthrops with a crust of microscleres at the surface.

Spicules: calthrops (no dichocalthrops) and sanidasters.

Calthrops (Figs 8B, 9B–C), often with bifid or bluntly rounded cladi, occasionally five-claded, in a wide size range but upper size relatively large, cladi length and thickness 92–*299.8*–426 × 8–*38.6*–55 µm (in holotype) and 36–*224.9*–480 × 6–*28.6*–60 µm, cladomes 126–*462.6*–618 µm (holotype) and 61–*300.4*–810 µm (paratype).

Sanidasters (Figs 8C, 9D), relatively uniform in size and spination, relatively small: 11–12.6–17 µm (holotype) and 12–*17.3*–21 µm (paratype).

####### Etymology.

Named after the locality of the holotype.

####### Habitat.

Consolidating coarse limestone fragments on rubble bottoms.

####### Distribution.

Senegal, Mauritania, possibly (Eastern) Mediterranean.

####### Remarks.

The new species was formerly identified as Dercitus (Stoeba) plicatus, but it differs clearly by the much larger upper size of the calthrops and the apparent absence of dichocalthrops. There are some discrepancies between the two type specimens in the average size and thickness of the spicules, but in view of the generally large size variation this is considered intraspecific variation. It is likely that Lévi’s Senegal record of Dercitus plicatus also fits with this species, as it was similarly white coloured, and also lacked dichocalthrops. The size of the cladi of the calthrops were given as maximum 250 × 30 µm which is clearly larger than in Mediterranean Dercitus plicatus and within the range of our new species.

The ZMA collection contains a tiny fragment of a specimen from the Aegean Sea donated by E. Voultsiadou (ZMA 21697) which has calthrops (Fig. 9E) with cladi up to 295 × 48 µm (cladomes up to 475 µm), and which lacked dichocalthrops. Sanidasters similar in size and shape to those of Dercitus (Stoeba) senegalensis sp. n., especially to the paratype. Apart from the wide geographic separation, this fits with our new species.

The record of Pansini (1987) of Dercitus plicatus from the Alboran Sea possibly also belongs to this species as it was noted to lack dichotriaenes. However, since no further data were provided this remains undecided.

###### Dercitus (Stoeba) verdensis sp. n.

urn:lsid:zoobank.org:act:F5098A5C-CED8-43DE-8EF1-A0401AAFE64A

Figs 10A–D

Dercitus plicatus ; Van Soest 1993: Table 2, pars (not: Dercitus plicatus sensu Schmidt 1868).

####### Material examined.

Holotype ZMA Por. 07521b, Cape Verde Islands, São Nicolau, Branco, 16.6667°N; 24.7167°W, dredged from 98 m, coll. R.W.M. Van Soest, HMS ‘Tydemann’ CANCAP VII Expedition stat. 156, 5 September 1986.

####### Description

(Fig. 10A). Encrusting sponge, agglutinating and consolidating limestone fragments (dead corallines and serpulids), pale yellow-coloured, with cartilaginous consistency. Growing together with a yellow-coloured Chaetodoryx sp. Size of agglutinated mass approx. 4×2 cm.

Skeleton: a confused mass of dichocalthrops with a thin crust of sanidasters.

Spicules: dichocalthrops (no calthrops), sanidasters.

Dichocalthrops (Fig. 10B) relatively large, regularly shaped, with limited size variation, protocladi more or less uniform in smaller and larger spicules, but deuterocladi longer in larger ones: protocladi 42–*48.2*–56 × 9–*21.6*–35 µm, deuterocladi 23–*106.8*–204 × 4–*17.2*–29 µm, rhabdomes 71–*170.6*–252 × 8–*21.0*–31 µm, cladomes 132–*294.6*–475 µm.

Sanidasters relatively small, little variation in spination and ornamentation, 11–*13.3*–16 µm.

####### Etymology.

Named after the type locality.

####### Habitat.

Deeper water, consolidating coarse sediment.

####### Distribution.

So far with certainty known only from the type locality. Possibly, Topsent’s 1928 record of Dercitus plicatus from Boavista also belongs to this species.

####### Remarks.

Although the specimen was originally identified with Dercitus plicatus, there are two compelling differences with that species: (1) there are no calthrops, whereas these are the dominant spicule in Dercitus (Stoeba) plicatus, and (2) the size of the dichocalthrops, especially the length of the deuterocladi, is up to twice that of Dercitus (Stoeba) plicatus.

###### Dercitus (Stoeba) extensus

(Dendy, 1905)

Figs 11A–D

Stoeba extensa Dendy 1905: 70, pl. V fig. 1; ? Vacelet and Vasseur 1971: 70, fig. 13.
Halina plicata ; Thomas 1970: 207, fig. 9.

####### Material examined.

BMNH 1907.2.1.5a, two slides from type, R.N. 167, Gulf of Mannaar, Sri Lanka.

####### Description

(partially from Dendy, 1905). Encrusting extensively over calcareous algae and filling crevices, 4.5×3.3 cm in lateral expansion. Surface smooth, no apertures. Colour pale grey (alcohol). Consistency tough, fleshy. Dendy mentions the presence of large cells with inclusions (cystencytes) of 60 µm.

The two slides contain thin cross sections of the peripheral regions.

Skeleton (Fig. 11A): irregular mass of short-shafted triaenes covered by a thin ectosomal crust of microscleres.

Spicules: megascleres predominantly dichocalthrops, but also normal calthrops or calthrops with one or two bifid cladi, sanidasters.

Calthrops (Fig. 11C), cladi 57– *87.0*–123 × 8–*14.9*–23 µm, cladomes 108–*158.9*–210 µm.

Dichocalthrops (Fig. 11B), protocladi 39–*46.9*–54 × 5–*15.5*–21 µm, deuterocladi 8–*37.9*–61 × 4–*11.1*–18 µm, rhabds 24–*65.6*–124 × 5–*15.1*–27, cladomes 84–*155.2*–192 µm.

Sanidasters (Fig. 11D), oxea-like (Dendy: ‘microxeas’), 14–*19.6*–26 × 1–*1.55*–2 µm.

####### Habitat.

Encrusting calcareous algae at depths of 18–63 m.

####### Distribution.

Sri Lanka, ?Madagascar.

####### Remarks.

The spicule complement reminds of Dercitus (Stoeba) pauper Sollas (1902), but the cladi and rhabds of the triaenes in this species are distinctly thinner (5–27 µm in *extensus*, 3–10 µm in *pauper*), precluding synonymy of the two species for the time being, until variation of these features is better established.

It is likely that the record of Halina plicata of Thomas (1970) belonged to the present species as most descriptive remarks fit except for the upper size of the cladome (‘chord’) of the dichocalthrops which was quoted as 330 µm.

Vacelet and Vasseur (1971) reported this species from Madagascar. Live colour was noted as black, which was maintained in alcohol. Dichocalthrops measurements provided were: protocladi 20–40 µm, deuterocladi 60–80 µm, rhabds 150–180 µm. Sanidasters were 25–30 µm. No mention of calthrops. No further spicule data. The colour difference, lack of calthrops, and slight differences in spicule sizes and shapes render it doubtful whether this material belongs to Dercitus (Stoeba) extensus.

###### Dercitus (Stoeba) pauper

Sollas, 1902

Dercitus pauper Sollas 1902: 218, pl. XVI fig. 1.

####### Material examined.

None. Holotype specimen, collected by R. Evans from Great Redang Island, Malaysia, could not be found in BMNH (2009).

####### Description

(from Sollas, 1902). Sponge encrusting a dead coral fragment, 50×5×1–2 mm, surface smooth, shining; no oscules. Colour pink.

Spicules: Megascleres dominated by small dichocalthrops, but there are also small calthrops.

Calthrops have cladi of 60–70 × 3 µm (computed cladomes 90–115 µm diameter).

Dichocalthrops with protocladi 50–60 × 10 µm, deuterocladi 30 µm and rabdome 80 µm (computed cladomes 160–180 µm in diameter).

Sanidasters long and thin, with oxea-like shape: 15–20 × 1 µm.

####### Habitat.

Intertidal, occurring on dead coral fragments.

####### Distribution.

So far only known from Great Redang Island, one of the coral islands of the state of Trengganu at approx. 5°N on the E coast of Malaysia.

####### Remarks.

By the small size of the dichocalthrops this species appears similar to Dercitus (Stoeba) occultus (see below), but that species lacks calthrops, and details of spiculation appear different (longer protocladi and shorter deuterocladi in Dercitus (Stoeba) pauper). Dercitus (Halinastra) sibogae sp. n. (see below) also has small dichocalthrops, but this likewise lacks calthrops and the microscleres are differentiated in long and thin and short and fat sanidasters, not reported for Dercitus (Stoeba) pauper. All three species are tropical shallow-water sponges occurring in West Pacific – East Indian Ocean waters.

###### Dercitus (Stoeba) occultus

Hentschel, 1909

Dercitus occultus Hentschel 1909: 352, text-fig. 1.

####### Material examined.

None. The type material collected by Michaelsen & Hartmeyer from Shark Bay, West Australia could not be found in ZMB (listed as a slide ZMB 4492 in Hooper and Wiedenmayer, 1994: 324).

####### Description

(from Hentschel, 1909). Endolithic within corals, filling tubular cavities of 1–2 mm diameter which connect to the outside in only a few places. Colour (in alcohol) a deep brown. The skeleton consists of dichocalthrops and sanidasters.

Dichocalthrops relatively small, protocladi 20–28 µm, deuterocladi 50–92 µm, rhabd 86–105 × 12–18 µm (computed cladome diameter 130–230 µm).

Sanidasters 13–21 × 1.5 µm. Spines are described as strongly developed and irregular, but only tiny drawings of these spicules are provided, which only show elongated rhabd shapes.

####### Habitat.

An endolithic species from shallow coral reefs (0.5–3.5 m).

####### Distribution.

West Australia, Shark Bay, shallow water.

####### Remark.

In spicule size this species appears close to Dercitus (Halinastra) sibogae sp. n. (see below), but that species has both ‘normal’ and compressed sanidasters, and it is epilithic. Details of the cladi lengths of the megascleres are also different.

The only other species of Dercitus (Stoeba) from Australian waters, Dercitus (Stoeba) xanthus Sutcliffe et al. 2010, differs sharply from the present species in the absence of dichocalthrops, in stead of which there are exclusively three-claded calthrops megascleres.

###### Dercitus (Stoeba) fijiensis sp. n.

urn:lsid:zoobank.org:act:7C305768-7C98-4417-A603-624E25D86FE9

Figs 12A–C

Halina plicata ; Tendal 1969: 34, figs 2A–B.

####### Material examined.

Holotype ZMUC unnumbered, Fiji Islands, Vitu Levu, Suva Harbour, coll. T. Wolff, 18 May 1965.

####### Description.

According to Tendal (1969) the sponge formed an irregular mass of 2×5×4 mm, situated in a crevice in a dead piece of coral. Smooth surface. Colour dark grey, oscules and pores not visible. Consistency hard. The material available to us consisted of a tiny fragment, approx. 1 mm3, half of which was sacrificed for SEM and a residue spicule slide.

Skeleton: unknown but presumably confused, with an ectosomal cover of microscleres.

Spicules: calthrops and dichocalthrops megascleres in approximately equal quantities, sanidasters.

**Figure 12.**
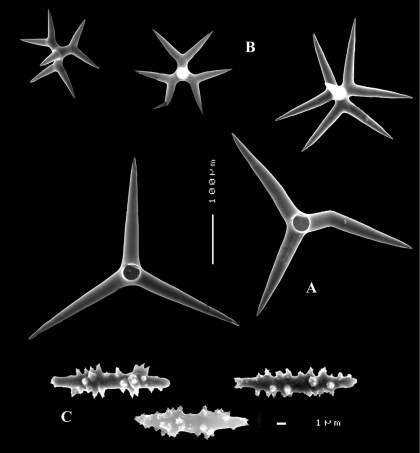
Dercitus (Stoeba) fijiensis sp. n., holotype from ZMUC, **A** calthrops **B** dichocalthrops, with type characteristic of the species at the right **C** sanidasters.

Calthrops (Fig. 12A), with cladi occasionally crooked or bifid at the apex, size relatively uniform with few smaller spicules, cladi 96–*222.7*–258 × 19–*31.2*–37 µm, cladome 186–*347.1*–420 µm.

Dichocalthrops (Fig. 12B), with smaller sizes normal shaped, but characteristically with very short protocladi and long conical deuterocladi when larger, protocladi 19–*26.5*–30 × 13–*25.2*–42 µm, deuterocladi 55–*112.5*–192 × 9–*20.2*–31 µm, rhabdomes 58–*84.6*–132 × 11–*13.6*–21 µm, and cladomes 129–*248.4*–361 µm.

Sanidasters (Fig. 12C), relatively thick, pointed-fusiform at low magnifications, in SEM mostly with blunt endings, relatively uniform in size and shape, 15–*16.9*–21 × 3–*3.6*–4 µm.

####### Etymology.

Named after its type locality.

####### Habitat.

Shallow-water, in crevices in dead corals.

####### Distribution.

Known only from the Fijian type locality.

####### Remarks.

The distinctive feature of this new species, apart from details of spicule sizes, is the characteristic shape of the larger dichotriaenes with very short protocladi and long conical deuterocladi (represented most clearly in the dichocalthops at the right of Fig. 12B). Tendal (1969) assigned this material to Dercitus (Stoeba) plicatus (as Halina), but this Mediterranean species has much smaller dichocalthrops (cladomes only up to approx. 200 µm) in a lower quantity than the calthrops, and is white in colour. Tendal’s measurements of the spicules differs slightly from our measurements. We did not see any sanidasters thinner than approx. 3 µm, whereas Tendal mentions 1–4 µm in thickness and protocladi of the dichocalthrops were never longer than 30 µm, whereas Tendal gives 24–66 µm. Possibly, the limited size of our fragment and the few spicules we could measure explains the differences. Even with Tendal’s measurements included, the differences with Dercitus (Stoeba) plicatus in the size of the dichocalthrops and calthrops remain clear. Furthermore, Tendal stressed that the deuterocladi are always longer than the protocladi and gave an excellent drawing of the characteristically shaped dichocalthrops. Thus the differences with Dercitus (Stoeba) plicatus are distinct and unambiguous.

The species closest to Dercitus (Stoeba) fijiensis sp. n. is probably Dercitus (Stoeba) extensus from Sri Lanka. Colour and other macroscopic features appear similar, as is the shape of the microscleres. Calthrops and dichocalthrops are slightly smaller (cladomes of both approx. 200 µm, against up to 400 and 300 µm respectively in Dercitus (Stoeba) fijiensis sp. n.). The characteristic shape of the dichocalthrops of Dercitus (Stoeba) fijiensis sp. n. is not found in Dercitus (Stoeba) extensus.

Dercitus (Stoeba) occultus Hentschel (1909) has its dichocalthrops shaped rather similarly to Dercitus (Stoeba) fijiensis sp. n. but cladome size is distinctly smaller; moreover this species lacks calthrops.

Other species with a complement of both calthrops and dichocalthrops, Dercitus (Stoeba) lesinensis, Dercitus (Stoeba) dissimilis, Dercitus (Stoeba) pauper, Dercitus (Stoeba) reptans, and Dercitus (Stoeba) bahamensis sp. n. likewise differ in (dicho-)calthrops size and shape.

###### Dercitus (Stoeba) reptans

Desqueyroux-Faúndez & Van Soest, 1997

Dercitus reptans Desqueyroux-Faúndez and Van Soest 1997: 402, Figs 50–52.

####### Material examined.

Holotype USNM 43170, Galapagos Islands, Albemarle Island, 0–3 m, 0.25°S; 91.4833°W, coll. Anton Bruun Cruise 16–66139, 25 May 1966 (slides and fragments of the holotype are present in MHNG, nr. 18963). Paratype ZMA Por. 11215, three fragments from the same locality.

####### Description

(from Desqueyroux-Faúndez and Van Soest 1997). Creeping branches encrusting corals. Surface micronulose, no oscules, whitish pink (preserved condition).

Skeleton: thick ectosomal crust of microscleres. Choanosomal skeleton confused mass of megascleres.

Spicules: calthrops, dichocalthrops, sanidasters. Some spicule types are remeasured to provide additional data and to correct a remarkable error in the original description.

Calthrops robust, variable in size, cladi 344–*484*–648 (remeasured 39–648) x 16–*27*–50 µm (remeasured 6–50 µm) (cladome up to 670 µm, remeasured 60–680 µm). The smaller calthrops category reported here is rare.

Dichocalthrops, or short-shafted dichotriaenes, small, cladome 156–*164*–170 µm (remeasured 141–*154.5*–180 µm), individual cladi up to 90 × 8–9 µm (remeasured: protocladi 27–*33.8*–36 × 9–*10.4*–12 µm; deuterocladi 35–*47.2*–69 × 6–*8.2*–11 µm; rhabds 57–*74.5*–106 × 9–*11.0*–12 µm).

Sanidasters: short and slim, with blunt apices. Remarkably, they were quoted to be 15–74 × 2–8 µm in size, but this is an obvious error. Remeasured they appear to be highly uniform in size and shape: 9–*12.8*–16 × 1–*1.9*–2.5 µm, which is also in accordance with the size and shape of the figured sanidaster (fig. 52).

####### Habitat.

Shallow water.

####### Distribution.

Only known from the Galapagos Islands.

####### Remarks.

By its large calthrops this species stands out among Dercitus species with a complement of small dichocalthrops. None of the extant species are morphologically close.

###### Dercitus (Stoeba) bahamensis sp. n.

urn:lsid:zoobank.org:act:AC0937B4-F5ED-406D-A4DC-E6DF6E331202

Figs 13 A–E

Dercitus  sp.; Kohmoto et al. 1988: 85.

####### Material examined.

Holotype HBOI 23–VIII–85–1–49, (HBOM 003.00040), with schizoholotype fragment ZMA Por. 07782, Bahamas, New Providence Island, NW of Goulding Cay, 25.025°N; 77.583°W, 210 m, coll. K. Rinehart in the Johnson SeaLink submersible, 23 August 1985, don. S.A. Pomponi and M.C. Díaz.

####### Description.

**Figure 13.**
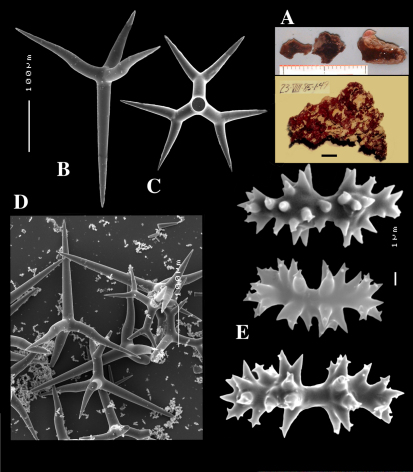
Dercitus (Stoeba) bahamensis sp. n., **A** schizoholotype ZMA Por. 07782 and holotype HBOI 23–VIII–85–1–49 (photo J. Reed, scale bar 1 cm), from Bahamas, New Providence Island **B** calthrops **C** dichocalthrops **D** overview of spicules **E** various sanidasters.

Slippery smooth encrustation on dead coral (Fig. 13A); the present material (upper part of Fig. 13A) consists of three fragments of 12×10, 8×8 and 8×5 mm, of approx. 1–2 mm in thickness. The holotype (lower part of Fig. 13A) is a larger fragment of 7×6 cm (not examined). Colour bright red when alive, darker red in alcohol. Consistency gum-like, easy to cut.

Skeleton: a confused mass of short-shafted triaenes and sanidasters.

Spicules: calthrops-like short-shafted triaenes, dichocalthrops and sanidasters.

Calthrops (Figs 13B, D) showing a clear rhabd, longer than the cladi, which are frequently curved or undulated or bifid. Cladi 138–*166.7*–186 × 12–*22.5*–28 µm, cladomes 207–*266.4*–330 µm in diameter, rhabds 230–*245.9*–270 × 24–*27.2*–30 µm.

Regular symmetrical dichocalthrops (Figs 13C–D) occur less frequently; protocladi 18–*29.0*–35 µm and deuterocladi 57–*68.1*–105 × 12–*14.1*–18 µm, rhabdomes 70–*141.0*–180 × 12–*20.7*–30 µm, cladomes 143–*197.9*–266 µm.

Sanidasters (Fig. 13E) have an overall short and squat shape. They tend to be amphiaster-like with frequently more heavily spined apices; spines usually with secondary spines. Size 12–*13.3*–15 × 2–*2.85*–3.5 µm.

####### Etymology.

Named for its type locality.

####### Habitat.

Collected by submersible at 210 m depth.

####### Distribution.

Known only from the Bahamian type locality.

####### Remarks.

This is a Dercitus (Stoeba) species similar to Brazilian Dercitus (Stoeba) latex (Moraes & Muricy, 2007) in shape, colour and consistency. It differs clearly in possessing dichocalthrops and larger unequal-claded calthrops, and the sanidasters are also clearly shaped and sized differently (long and thin with simple spines in Dercitus (Stoeba) latex). The Bonaire Dercitus (Stoeba) specimen described below differs in being insinuating, lacking dichocalthrops and also having thin sanidasters with undivided rays. Dercitus (Halinastra) lutea (Pulitzer-Finali, 1986, see below) is yellow, lacks dichocalthrops and has compressed sanidasters in its spicule complement. Dercitus (Halinastra) arubensis sp. n. (see below) differs from the new species in lacking dichocalthrops and possessing thick smooth microrhabds.

###### Dercitus (Stoeba) latex

(Moraes & Muricy, 2007)

Stoeba latex Moraes and Muricy 2007: 1388, figs 2–3.

####### Material examined.

None.

####### Holotype.

MNRJ 8559, Belmonte Islet Wall, São Pedro e São Paulo Archipelago, Brazil, 0.9167°N; 29.35°W, 30 m depth, coll. F. Moraes, 15 July 2004.

####### Description

(from Moraes and Muricy 2007). Thickly encrusting to massive, reddish- brown (darker in preservation), surface smooth, ‘stretched’; oscules 0.5–3 mm, size up to 10×20 cm. Consistency firm, rubbery. Ectosome provided with spherulous cells and sanidaster microscleres, choanosome compact, with randomly dispersed calthrops.

Spicules. Calthrops 42–212 × 7.5–25 µm. Sanidasters 10–15 × 1 µm.

####### Habitat.

Vertical walls and overhangs, 4–30 m.

####### Distribution.

Offshore archipelagoes to the NE of Brazil.

####### Remarks.

The red colour is shared with Dercitus (Stoeba) bahamensis sp. n., but this has dichocalthrops and the spicule sizes are also different. Below we report an undescribed Dercitus (Stoeba) spec. from Bonaire for which insufficient material was left after making preparation. It differs in habit (insinuating) and spicule size (calthrops cladi smaller, only up to 186 ×16 µm, and sanidasters slightly larger, up to 18 × 1.5 µm). Cladi of calthrops of the Bonaire material are frequently bifid and some ?auxiliary oxeas were present in the slides. Nevertheless, similarities are close enough to reckon with the possibility that the two are conspecific members of a variable species.

###### Dercitus (Stoeba) syrmatitus

De Laubenfels, 1930

Dercitus syrmatitus De Laubenfels 1930: 26; 1932: 38, fig. 17.
Stoeba syrmatitus ; Lee et al. 2007: 213.

####### Material examined.

None.

####### Holotype.

USNM 21438, United States, California, Laguna Beach, coll. M.W. De Laubenfels, 26 March 1929.

####### Description

(from De Laubenfels, 1932). Consolidating sandy substrate, shape ‘amorphous’, colour ‘drab’, surface smooth, consistency slimy. Size of parts containing the characteristic spiculation 0.2–2 mm. Skeleton of interior ‘packed’ with megascleres and microscleres.

Spicules: calthrops and sanidasters.

Calthrops, small, often reduced to ‘tripods’, cladi 25–*65*–80 × 3–*8*–10 µm.

Sanidasters variable in spination, often concentrated in two areas of the microsclere to approach an amphiaster condition, but many are irregular, endings blunt, 8–12 µm.

####### Habitat.

Intertidal.

####### Distribution.

California.

####### Remarks.

De Laubenfels (1932) mentions the possible presence of toxas, judged to be foreign. Lee et al. (2007) provide SEM photos of the spicules. The species stands out by its small calthrops, which is only shared with Dercitus (Stoeba) xanthus, but in that species there are no normal calthrops, only three-claded ones, and the sanidasters are considerebaly longer.

###### Dercitus (Stoeba) xanthus

Sutcliffe, Hooper & Pitcher, 2010

Dercitus xanthus Sutcliffe, Hooper and Pitcher 2010: 6, figs 4–5.

####### Material examined.

None.

####### Holotype.

QMG329976 (SBD513022), south of Rock Cod Shoal, off Gladstone, Great Barrier Reef, 23.725°S; 151.6647°E, 34 m depth, epibenthic sled, coll. FRV ‘Lady Basten’, 20 September 2004.

####### Description

(from Sutcliffe et al. 2010). A thin sponge agglutinating biogenic rubble such as remains of worm tubes, gastropods and bivalves. Usually fist-sized or smaller. Live color red to yellow, surface uneven, no visible oscules.

Spicules: three-claded calthrops and sanidasters.

Calthrops small, divisible in two size classes with means of approximately 25 and 72 µm. Only 20% of the 163 specimens recorded possessed calthrops, usually in high densities, in the remaining 80 % these spicules were lacking. Sanidasters universally present in all specimens, displaying a wide variation in length and width, 10–20 × 1–2.5 µm, densely spined with relatively short spines up to 1 µm.

####### Habitat.

Sandy bottoms between 16 and 86 m depth.

####### Distribution.

Great Barrier Reef, occurring over the entire range.

####### Remarks.

As Sutcliffe et al. (2010) point out, there is only one species among the Dercitus s.l. species that is similar, viz. Dercitus (Stoeba) syrmatitus, sharing the possession of three-claded calthrops, the small size of the calthrops and the agglutinating habit. The differences are nevertheless quite clear and compelling, the rarity of the megascleres, the lack of normal fourc-claded calthrops and the distinctly larger sanidasters.

The only other Australian record of the genus, Dercitus (Stoeba) occultus, from West Australia, differs sharply in having exclusively dichocalthrops megascleres.

###### Dercitus (Stoeba)

Stoeba  sp. ? Vacelet and Vasseur 1971: 70, fig. 14.

####### Material examined.

None.

####### Description

(from Vacelet and Vasseur 1971). Encrusting the underside of coral blocks, 2–3 cm2 in expansion; consistency soft; live colour yellow, white in alcohol.

Spicules: calthrops, sanidasters, ?strongyles.

Calthrops with irregular cladi, 50–250 × 15–25 µm.

Sanidasters short and fat, 12.5 × 2.5 µm.

Strongyles, probably foreign, long and thin 375–400 × 3–5 µm.

####### Habitat.

Shallow reefs.

####### Distribution.

Known only from the Tuléar area, Madagascar.

####### Remarks.

Live colour and the details of the spicules make it likely that this is an undescribed species. The strongyles are considered foreign as the authors themselves suggested.

###### Dercitus (Stoeba)

Dercitus  sp. Kobluk and Van Soest 1989: 1211.

####### Material examined.

ZMA Por. 08985, Netherlands Antilles, Bonaire, W coast, near Kralendijk, in reef caves, 12–43 m, 12.15°N; 68.278°W, 1984, coll. D.R. Kobluk.

####### Description.

Insinuating in coral crevices, in reef caves between 10 and 40 m depth. The available sponge material has been ‘used up’ entirely for the initial slides from which the genus identification (as Dercitus) was made. Properties of the sponge and structure of the skeleton cannot be accurately described. The skeleton is a confused, dense mass of calthrops megascleres on the outside covered by a layer of microscleres. These are also common throughout the sponge body. Spicules are simple calthrops and sanidasters.

Calthrops regular, with four equal cladi, which may occasionally be curved; endings occasionally bifid or irregularly terminally knobbed, but no true dichotriaene nor triactine modifications are present; sizes highly variable, cladi 39–*102.3*–186 × 4–*9.8*–16 µm, cladomes ranging 58–*159.7*–288 µm.

Sanidasters, thin, 12–*14.8*–18 × 0.5–*0.76*–1.5 µm.

####### Habitat.

In reef caves at the deeper parts of fringing reefs.

####### Remarks.

Kobluk and Van Soest (1989) reported a few oxeas of 600 µm length, which are here considered foreign, and ‘dichotriaene-like variations’ by which they meant the calthrops with bifid cladi, not to be confused with true dichocalthrops. Since no proper specimen was left, no SEM observations of the spicules of this species could be made. It is likely that it is an undescribed species. It differs from Central West Atlantic Dercitus (Stoeba) latex (Moraes & Muricy, 2007) in habit (encrusting in *latex)*, and spicule size (calthrops cladi larger, up to 212 × 25 µm, and sanidasters smaller, up to 15 × 1 µm). Dercitus (Stoeba) bahamensis sp. n. likewise is encrusting and it has dichocalthrops and thicker sanidasters (see above).

##### Sungenus Halinastra

De Laubenfels, 1936

Halinastra De Laubenfels 1936: 179.

###### Definition:

Dercitus with clear subdivision of the sanidaster microscleres into longer, thinner irregular sanidasters and shorter, fatter, compressed, often aster-like forms.

###### Type species:

Pachastrella exostotica Schmidt, 1868 (by original designation).

###### Key to the species of Dercitus (Halinastra).

**Table:** 

1	Live colour yellow turning to dark brown or black in alcohol	Dercitus (Halinastra) luteus
–	Live colour black	2
–	Colour in alcohol beige or orange brown, compressed sanidasters ovoid, not euaster-like	3
2	Sanidasters very long up to 42 × 10 µm	Dercitus (Halinastra) berau sp. n.
–	Sanidasters long and thin, up to 33 × 6 µm	Dercitus(Halinastra) japonensis sp. n.
–	Sanidasters relatively short and fat, up to 27.5 × 7.5 µm	Dercitus (Halinastra) exostoticus
3	Insinuating habit, exclusively calthrops megascleres, microscleres include peculiar microspined ovoid rhabds	Dercitus (Halinastra) arubensis sp. n.
–	Encrusting habit, exclusively dichocalthrops megascleres, microscleres two sanidaster shapes, long-and-thin and fat-compressed	Dercitus (Halinastra) sibogae sp. n.

###### Dercitus (Halinastra) exostoticus

(Schmidt, 1868)

Figs 14A–D

Pachastrella exostotica Schmidt 1868: 16, pl. III fig. 12; Keller 1891: 343, pl. 19 fig. 53, pl. 20 fig. 54.
Calthropella exostitus  (sic); Sollas 1888: 111.
Pachastrella (Pachastrella) exostotica ; Lendenfeld 1903: 75.
Halinastra exostotica ; de Laubenfels 1936: 179.
Stoeba exostita  (sic); Maldonado 2002: 156. Figs 2F–G.

####### Material examined.

Holotype, ZMB 287, Eritrea, Dahlak Archipelago, near Perim Island, Red Sea, 16.75°N; 40.05°E, 52 m, coll. Siemens.

####### Description.

**Figure 14.**
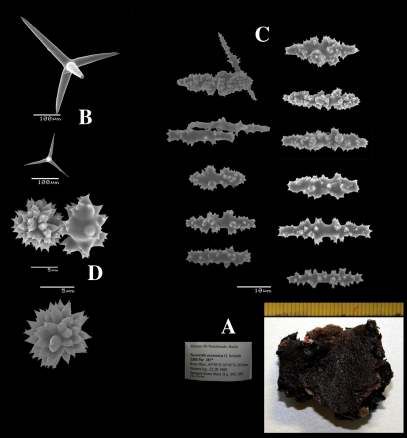
Dercitus (Halinastra) exostoticus, holotype ZMB 287, from Eritrea, Dahlak Archipelago, **A** habit **B** various calthrops **C** various sanidasters **D** various aster-like compressed sanidasters.

Black mass (in alcohol) of 3×3×0.5 cm (Fig. 14A). Surface irregular granulated, grooved, no apparent oscules. In cross section, only the surface region is darkly pigmented, but the choanosome is much lighter coloured. According to Keller choanocyte chambers are crowded in the lower parts of the choanosome, size 25 µm.

Skeleton: a confused, dense mass of calthrops with a cover of microscleres at the surface. A few oxeas of widely different sizes were noted by Keller, but these were limited between approx. 100–120 × 2.5 µm of a typical haplosclerid type, in the slide examined by us.

Spicules: calthrops, sanidasters, compressed sanidasters (pseudasters).

Calthrops (Fig. 14B), generally regularly shaped, in a wide size range, usually with one of the cladi slightly longer than the other three (short-shafted triaenes): 45–*128.2*–275 × 5–*18.2*–35 µm.

Sanidasters (Fig. 14C), variable in thickness and spination, but relatively uniform in length, generally ‘stocky’, 15–*23.1*–27.5 × 2.5–5*.3*–7.5 µm.

Compressed sanidasters (pseudasters) (Fig. 14D), globular, irregular in shape (Keller called these ‘Sterraster oder Sphaeraster’), 7.5–*10.5*–12.5 µm in diameter.

####### Habitat.

Deeper water, beyond the reefs

####### Distribution.

Known only from the Southern Red Sea.

####### Remarks.

Schmidt (1868) gave hardly any description (some vague remarks on the megascleres of Pachastrella monilifera grading into a statement on the Red Sea specimen), but the drawings of the microscleres show a sanidaster and a globular aster. Keller claims to have had access to Schmidt’s type specimen, but he faithfully reports that there is a discrepancy between Schmidt’s original label and the label of his redescribed specimen, e.g. the original collector was stated to be Ehrenberg by Schmidt, but Keller states it is Siemens, which is probably the correct one.

Maldonado (2002) assigned Pachastrella exostotica to the genus Stoeba and declared Halinastra a junior synonym of that genus. However, he possibly overlooked that Pulitzer-Finali’s (1986) Pachataxa lutea possesses similar spiculation as Pachataxa exostotica (see below). With at least two species sharing the peculiar aster-like compressed acanthorhabds, (and several further species with diversified aster shapes, see below) there is good reason to maintain Halinastra as a distinct taxon, proposed here to be a subgenus.

###### Dercitus (Halinastra) berau sp. n.

urn:lsid:zoobank.org:act:6960CBF1-D287-4513-A1C9-1777580A67F2

Figs 15A–B 16A–D 

####### Material examined.

Holotype RMNH 4256, Indonesia, Kalimantan, Berau region, Derawan Islands, depth 10 m, coll. N.J. De Voogd, field nr. BER105/140808/055, SCUBA, 14 August 2008.

####### Description.

**Figure 15.**
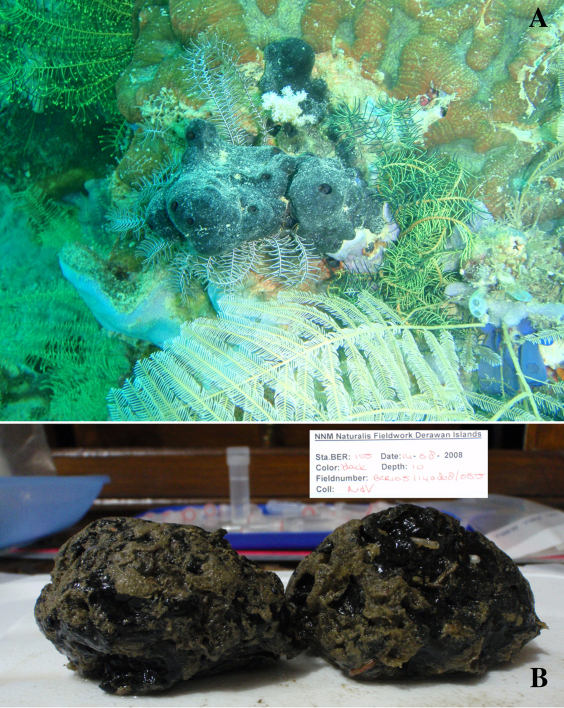
Dercitus (Halinastra) berau sp. n., holotype RMNH 4256, from Indonesia, Kalimantan, **A** photographed in situ **B** photographed just after collection (photos N.J. De Voogd).

**Figure 16.**
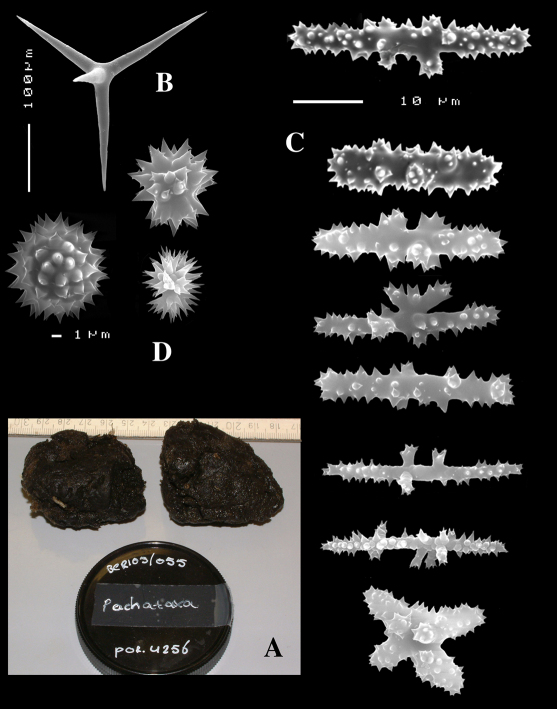
Dercitus (Halinastra) berau sp. n., holotype RMNH 4256, **A** Habit after preservation **B** calthrops **C** various sanidasters **D** various aster-like compressed sanidasters.

Blackish grey lobate mass (Fig. 15A), approx. 20×15×10 cm in size, surface in places speckled white by ?coral sand and at the base the specimen is encrusted by a bluish sponge (Haliclona). Oscules prominent on the summit of lobes, approx. 0.5 cm in diameter. Consistency firm, compressible. The preserved specimen is broken into two similar sized masses (Figs 15B, 16A). The black colour is maintained in alcohol and has strongly darkened the fluid and the labels.

Skeleton: difficult to study in the preserved condition due to the intense black colour, but structure is dense and unorganized, with a dense cover of microscleres at the surface.

Spicules: calthrops, sanidasters and compressed spheraster-like microscleres (pseudasters); a few oxeas are present, but these belong to the encrusting Haliclona.

Calthrops (Fig. 16B), generally regular in shape and cladi number, occasionally three-claded or five-claded; size highly variable, cladi 25–*151.4*–280 × 5–*17.2*–35 µm.

Sanidasters (Fig. 16C) of extreme variability in shape, long thin with short spines, thicker with prominent stubby spines and squat warty ones, with many intermediates; size 19–*29.1*–42 × 1–*5.1*–10 (spines 0.5–*2.2*–4.5 µm).

Compressed sanidasters (pseudasters) (Fig. 16D), globular or oval with very short rays (usually less than 0.5 µm), diameter 8–*11.4*–15 µm.

####### Etymology.

Named after the Berau region, East Kalimantan.

####### Habitat.

Shallow open reef localities.

####### Distribution.

So far known only from the type locality E of Kalimantan.

####### Remarks.

Despite the long distance between the locality of Dercitus (Halinastra) exostoticus (Red Sea) and the present locality, there are compelling similarities in shape, colour and spicule sizes between the new species and *exostoticus*. The major difference between the two is the length and robustness of the sanidasters (up to 42 µm in Dercitus (Halinastra) berau sp. n. against max. 27.5 µm in Dercitus (Halinastra) exostoticus). We also believe that the habit and the choanosome pigmentation will prove to be distinct in the two, but the absence of observations of living Dercitus (Halinastra) exostoticus precludes definite conclusions.

Dercitus (Halinastra) berau sp. n. differs from Dercitus (Halinastra) japonensis sp. n. (see below) in the more robust condition of the sanidasters, the more oval, not completely euaster-like condition of the compressed sanidasters of the Japanese specimens. Also the length of the calthrops cladi appears less in the Dercitus (Halinastra) japonensis sp. n. See also Table 3.

###### Dercitus (Halinastra) japonensis sp. n.

urn:lsid:zoobank.org:act:FF7DBEA7-C1DE-429A-A309-743FAAF8068F

Figs 17A–D 18A–C

Stoeba extensa ; Phuwapraisirisan et al. 2004: 2128

####### Material examined.

Holotype ZMA Por. 17646 (voucher fragment of a specimen used in natural products research) Japan, near Anami-oshima Island, 157–161 m, 28.873°N; 129.5532°E, coll. N. Fusetani, dredge, field nr. S01–111, 7 November 2003.

Paratype ZMA Por. 19889 (voucher fragment of a specimen used in natural products research), Japan, Kagoshima Prefecture, Ooshima-shinsone, 150 m, 28.8667°N; 122.55°E, coll. Y. Nakao, dredge, field nr. S01–118, 16 July 2001.

####### Description.

**Figure 17.**
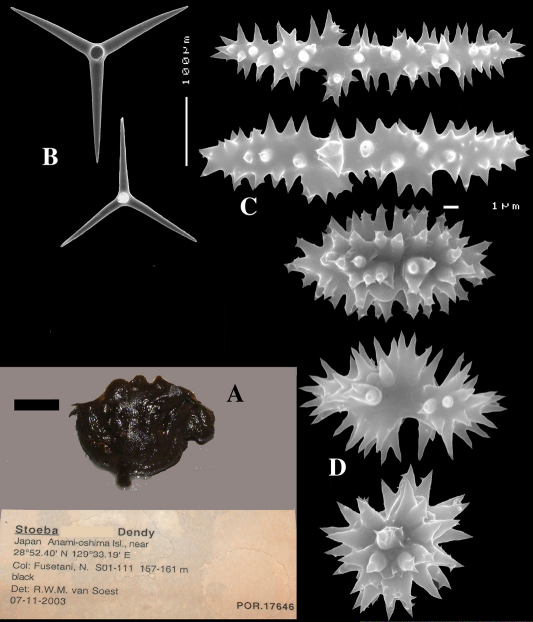
Dercitus (Halinastra) japonensis sp. n., holotype ZMA Por. 17646, from Japan, Amami-oshima Islands **A** habit (scale bar 1 cm) **B** calthrops **C** sanidasters **D** compressed sanidasters.

Holotype (Fig. 17A) is a blackish brown fragment of 3.5×3×1 cm (live colour also noted as black). Surface lumpy but smooth, shiny. Consistency firm, compressible.

Skeleton: at the surface a crust of microscleres, overlying a dense confused mass of calthrops.

Spicules: calthrops, sanidasters, compressed sanidasters (pseudasters).

**Figure 18.**
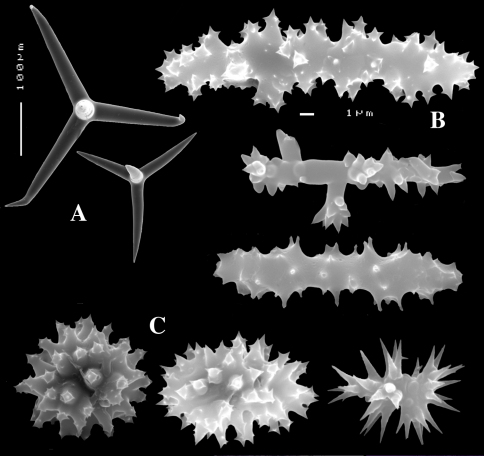
Dercitus (Halinastra) japonensis sp. n., paratype ZMA Por. 19889, from Japan, Ooshima-shinsone, **A** calthrops **B** sanidasters **C** compressed sanidasters.

Calthrops of holotype (Fig. 17B) equiangled, regular, 105–*170.8*–223 × 14–*20.7*–28 µm (cladomes 182–*286.3*–365 µm); of the paratype (Fig. 18A), equiangled, some with ‘hooked’ apices, 33–*177.2*–252 × 5–23.9–37 µm (cladomes 63–*319.0*–451 µm).

Sanidasters of holotype (Fig. 17C) long, relatively slim, 27–*28.8*–31 × 2.5–*3.9*–4.5 µm; of the paratype (Fig. 18B), similarly slim, 19–*25.7*–33 × 2–*4.5*–6 µm.

Compressed sanidasters of the holotype (Fig. 17D), mostly elongated-oval, only few are more or less globular, 7.5–*10.1*–15 µm; of the paratype (Fig. 18C), similarly oval or even shaped as ‘double’ asters, 7–*9.3*–14 µm.

####### Etymology.

Named after the Japanese type locality.

####### Habitat.

Dredged from deeper water, at approx. 150 m.

####### Distribution.

Both specimens came from nearby locations, so the distribution so far appears limited to offshore Japan.

####### Remarks.

It is with some hesitation that these two Japanese sponges are assigned to a new species, rather than to Dercitus (Halinastra) berau sp. n. The two species appear generally similar, but sanidasters are shorter and slimmer than in Dercitus (Halinastra) berau sp. n. and the compressed sanidasters are few and consist mostly of oval rather than globular shapes. Combined with the clearly deeper occurrence and the considerable geographic distance we believe it is justified to consider both distinct at the species level. The two are obviously closely related to Dercitus (Halinastra) exostoticus (see Table 3) with which they form a species complex.

**Table 3.** Spicule sizes of specimens similar to Dercitus (Halinastra) exostoticus.

Author	calthrops cladi	sanidaster	compressed sanidaster	locality
Dercitus (Halinastra) exostoticus				
Schmidt 1868	not given	not given	½ sanidaster size	Red Sea
Keller 1891	200×25	25	5–10	Red Sea
Remeasured here	45–275×5–35	15–27.5×2.5–7.5	7.5–12.5	Red Sea
Dercitus (Halinastra) berau sp. n.				
RMNH 4256	25–280×5–35	19–42×1–10	8–15	Indonesia
Dercitus (Halinastra) japonensis sp. n.				
ZMA Por. 17646	105–223×14–28	27–31×2.5–4.5	7.5–15	Japan
ZMA Por. 19889	33–252×5–37	19–33×2–6	7–14	Japan

###### Dercitus (Halinastra) luteus

(Pulitzer-Finali, 1986)

Figs 19A–D 20A–E

Pachataxa lutea Pulitzer-Finali 1986: 74, figs 8–9.

####### Material examined.

ZMA Por. 10100, Netherlands Antilles, Curaçao, Boca Sami, 20 m, 12.142°N; 69.007°W, coll. P. Willemsen nr. C92 A20, July 1992.

ZMA Por. 14080, Netherlands Antilles, Curaçao, near Carmabi, in reef cave, 10 –20 m, 12.124°N; 68.974°W, coll. I. Wunsch nr. 84, January 1999.

ZMA Por. 21692 (ex MNRJ 6678), Brazil, Das Rocas Atoll, Fenda, 3.8584°S; 33.8057°W, coll. E. Hajdu, M.V. de Oliveira & U.S. Pinheiro.

####### Description.

**Figure 19.**
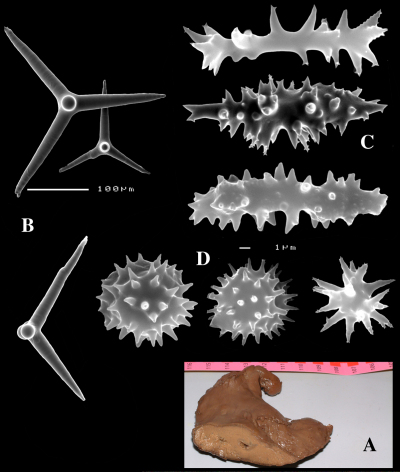
Dercitus (Halinastra) luteus, ZMA Por. 10100, from Netherlands Antilles, Curaçao, **A** habit **B** various calthrops **C** sanidasters **D** aster-like compressed sanidasters.

The larger specimen (10100) is a thick liver-like mass (Fig. 19A) with flat smooth surface, 8×6×3 cm in size, consistency compact, cheesy to rubbery. Live colour bright yellow, in alcohol dark brown. The preserved specimen shows a darker outer rim of 1–2 mm in thickness over a lighter coloured choanosome. This is not evidence of a cortex, it merely shows that the post-mortem discolouration does not penetrate evenly throughout the sponge. Our specimen conforms in all macroscopical aspects to Pulitzer-Finali’s type. The second specimen is smaller and thinner, approx. 1×1×0.2 cm, dark glistening red, with a slightly bumpy surface. The Brazil specimen is sized 4×4×4 cm, cut off from a much larger sponge.

Skeleton: the ectosome has a dense layer of sanidasters and ‘spherasters’ carried by a similarly dense mass of calthrops. In the choanosome the calthrops and sanidasters are less densely and confusedly distributed. There is no zonation or other structural arrangement of the skeleton.

Spicules: calthrops, sanidasters and compressed spheraster-like sanidasters (pseudasters).

Calthrops (Fig. 19B) are often three-claded, or even irregularly two-claded, some of the cladi may be straight, others are bifid terminally or wavy, of unequal length, stunted, irregular, unevenly curved. No dichocalthrops. Cladi 60–*120.2*–174 × 9–*14.9*–21 µm (measurements of both specimens combined), cladomes 102–*193.9*–264 µm.

Sanidasters (Fig. 19C) highly variable in ornamentation and spination, spines not obviously concentrated at the apices, relatively more frequently concentrated in the middle region, 12–*19.6*–26 × 1–*2.3*–4 µm.

Compressed sanidasters (Fig. 19D) are peculiar, irregular, multirayed, with a very thick center and very short rays, which appear spine-like rather than issuing from a common center, 5–*7.7*– 9 µm.

**Figure 20.**
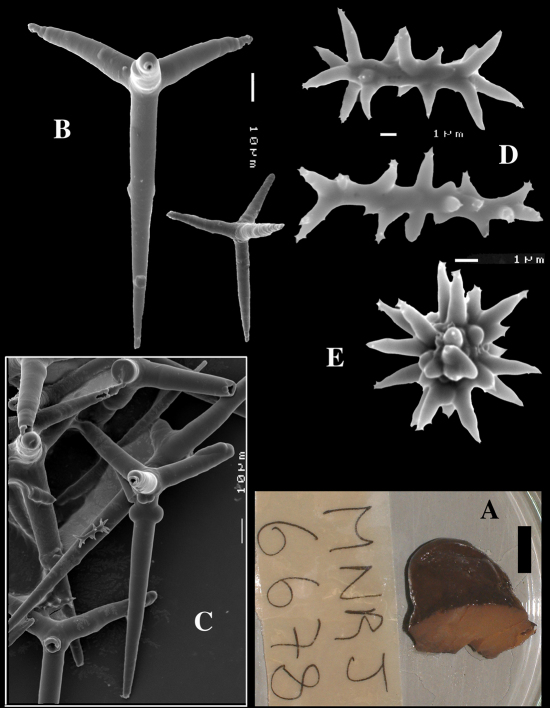
Dercitus (Halinastra) luteus, deviating specimen ZMA Por. 21692 (ex MNRJ 6678), from Brazil, Das Rocas Atoll, **A** habit (scale bar 1 cm) **B** short-shafted triaenes **C** overview of spicules **D** sanidasters **E** aster-like compressed sanidaster.

####### Habitat.

Open substrate in deeper parts of the reef, more shallow in caves.

####### Distribution.

Widespread Central West Atlantic: Puerto Rico, Southern Caribbean, Northeastern Brazil.

####### Remarks.

The Curaçao material appears indistinguishable from the Puerto Rico type material (MSNG 47681, La Parguera, Puerto Rico, 20–25 m) in overall morphology and spicules. Pulitzer’s material measures 7×5×1.5 cm, turning from light yellow to dark brown in alcohol. Consistency was described as fleshy, firm, compressible. Cladi of the calthrops were given as 70–180 µm (60–174 in our material), sanidasters (called microrhabds by Pulitzer) were given as 8–26 × 3–6 µm (12–26 × 1–4 µm in our material). Compressed sanidaster size was not given by Pulitzer but from his drawings can be estimated to be about 7–8 µm (5–9 µm in our material).

Through the courtesy of Prof. Hajdu we received a fragment (Fig. 20A) of a fleshy specimen from the oceanic island groups east of North East Brazil, originally bright yellow, now dark brown in alcohol and staining the alcohol considerably. The fragment showed several differences with the Curaçao and Puerto Rico specimens (see also Table 4): calthrops (Figs 20B–C) were often more like short shafted triaenes with one cladus considerably longer than the others; they were generally much more irregular and some appeared to have mesotriaene modifications. Length of cladi 33–*82*–123 × 4–*7.5*–11 µm. Sanidasters (Fig. 20D) were only 10–*14*–19 × 0.5–*1.1*–2 µm, and compressed sanidasters (pseudasters) (Fig. 20E) 5–*7.2*–9 µm. These differences may eventually lead to a distinction of a separate Brazilian species but for the time being we emphasize the similarities.

**Table 4.** Spicule data for specimens identification as Dercitus (Halinastra) Luteus.

*Author*	*calthrops cladi*	*sanidaster*	*compressed sanidaster*	*locality*
Pulitzer-Finali 1986	70–180××	8–26×3–6	7–8	Puerto Rico
ZMA 10100	60–174 ×9–21	12–26×1–4	5–9	Curaçao
ZMA 14080	80–130×15	18–21×3–4	7–9	Curaçao
ZMA 21692 (MNRJ 6678)	33–123×4–11	10–19×0.5–2	5–9	Oceanic islands Brazil

*‘Pachataxa’ lutea* does not belong to Calthropella (Pachataxa) for two reasons (see also below): (1) it possesses sanidasters, (2) there are no ataxasters, and (3) the spherasters do not resemble true euasters, they are compressed derivations of the irregularly spined sanidaster-like acanthomicrorhabds. In any case, they seem to be morphologically very close to the Red Sea species Dercitus (Halinastra) exostoticus Schmidt (1868 as Pachastrella) (see above).

###### Dercitus (Halinastra) arubensis sp. n.

urn:lsid:zoobank.org:act:90683880-683F-4EDB-B392-1B759B1AEDE3

Figs 21A–F 

####### Material examined.

Holotype ZMA Por. 08984, Netherlands Antilles, Aruba, Lagoon Boekoeti, underneath coral rubble, 0–1 m, coll. P. Wagenaar Hummelinck, nr. 1004, 29 December 1948.

####### Description.

**Figure 21.**
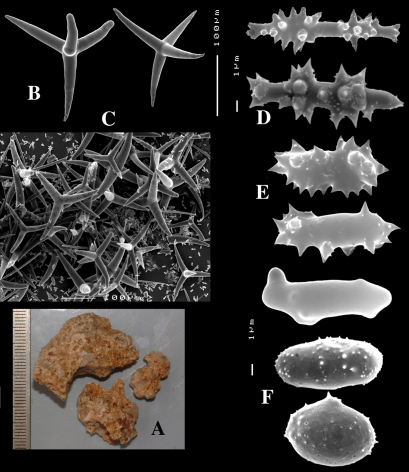
Dercitus (Halinastra) arubensis sp. n., holotype ZMA Por. 08984, from Netherlands Antilles, Aruba, **A** habit insinuating and encrusting coral rubble **B** various calthrops **C** overview of spicules **D** sanidasters **E** compressed sanidasters **F** smooth and spined microrhabds.

Small soft patches of a brown-red colour insinuating in three coral fragments (Fig. 21A), occupying spaces less than 5 mm in diameter. No remarkable macroscopical features.

Skeleton: Confused mass of calthrops and microscleres, no explicit structure.

Spicules: Calthrops, sanidasters and microrhabds.

Calthrops (Figs 21B–C) with gradually curved cladi, the apices of the cladi variously sharply pointed or blunt, occasionally displaying three-claded forms. Cladi: 60–*119.2*–174 × 9–*14.9*–21 µm, cladomes: 96–*192.7*–264.

Sanidasters (Fig. 21D) variable in shape and size, varying from thin amphiaster-like forms with clusters of spines at the ends or at least separated in the middle by a stretch with few spines, to fusiform or fat simple spined forms, size 16–*20.8*–26 × 1–*2.1*–3.5 µm.

Microrhabds (Figs 21F) short and thick, almost smooth, with only scattered very short spines, 5–*7.7*–9 × 3–4 µm. The microrhabds very likely derive from the sanidasters as there are some stages (Fig. 20E) which appear intermediate.

####### Etymology.

Named after the holotype locality.

####### Habitat.

Shallow-water, under coral rubble.

####### Distribution.

So far known only from the island of Aruba, Southern Caribbean.

####### Remarks.

The microrhabds are unique among species of the genus Dercitus s.l. The genus Pachastrella also possesses short and thick acanthose microrhabds, but in that genus the microrhabds are much more regularly spined, not of the irregular type found in the present species. Furthermore, Pachastrella species possess oxeas as megascleres next to calthrops or short-shafted (dicho-)triaenes and various types of streptasters such as amphiasters and spirasters. Calthropella (Pachataxa) species (family Calthropellidae) have ataxasters which may look similar in shape, but in addition these possess euasters (see below).

###### Dercitus (Halinastra) sibogae sp. n.

urn:lsid:zoobank.org:act:24C3A89D-99AE-4C39-B1DB-E028D9516826

Figs 22A–D 

####### Material examined.

Holotype ZMA Por. 02220, Indonesia, Papua, 32 m, 1.7083°S; 130.7916°E, coll. Siboga Exped. Stat. 164, dredge, 20 August 1899.

####### Description.

**Figure 22.**
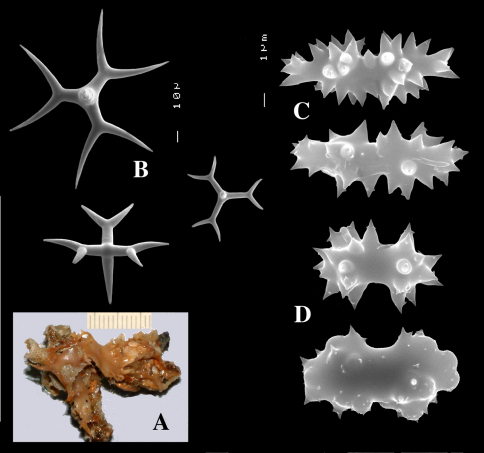
Dercitus (Halinastra) sibogae sp. n., holotype ZMA Por. 02220, from Indonesia, Papua, **A** habit **B** various dichocalthrops **C** sanidasters **D** compressed sanidasters.

Thin leathery-rubbery encrustation (Fig. 22A) cementing coral rubble and filling crevices in coral material. Colour (alcohol) pale orange brown. Surface smooth, not encrusted, no oscules apparent; interiorly with a different more mushy texture. Size 2.5×2×0.5 cm.

Skeleton: an ectosomal crust of microscleres overlying a mixture of microscleres and dichocalthrops embedded in largely organic choanosome with relatively low spicule density.

Spicules: dichocalthrops, sanidasters and compressed fat sanidasters.

Dichocalthrops (Fig. 22B) relatively small and delicate, with curved pointed deuterocladi; protocladi, rather uniform in length, more variable in thickness, 24–*26.5*–30 × 5–*8.0*–11 µm; deuterocladi variable in length, 12–*26.1*–39 × 3–*5.6*–9 µm; rhabd short and pointed, 36–75 × 6–9 µm; cladome 75–*101.1*–144 µm.

Sanidasters in two categories, long-and-thin (Fig. 22C) and short-compressed (Fig. 22D); the latter are ovoid, not compressed to the extent that they form asters. Thin sanidasters, 12–*13.9*–16 × 1.5–*2.05*–3 µm, short-compressed sanidasters 7–*11.0*–13 × 4.5–*5.6*–7.5 µm.

####### Etymology.

Named after the Dutch naval vessel HMS ‘Siboga’ which was instrumental to the 1899–1900 collection of a rich deep sea fauna from South East Asian waters.

####### Habitat.

Dredged from a sandy bottom with small limestone rubble and shells.

####### Distribution.

Known only from the type locality in Eastern Indonesia.

####### Remarks.

The present specimen was identified by Maurice Burton (who provisionally identified a large part of the Siboga sponge collection) as Stoeba extensa, but habit and colour differ from that of Dendy’s species, whereas the spicules are significantly smaller. Stoeba extensa has a dense mass of dichocalthrops but also calthrops which are absent from the present new species. The density of megascleres is also much different. The dichocalthrops appear similar to those of Dercitus (Stoeba) occultus (see above), and also the colour matches. However, that species has an endolithic growth form and the sanidasters are apparently not divisable in two shapes. The membership of Halinastra of this species rests on the differentiation of the sanidasters into two shapes, but proper pseudasters are not found. If the spicular variation of Dercitus (Stoeba) occultus and Dercitus (Halinastra) sibogae sp. n. will be demonstrated to overlap in a future study it might turn out the two are conspecific, but for the time being the emphasis is put on the differences.

#### Nominal Dercitus and Stoeba species excluded from the genus

##### Dercitus loricatus

Lebwohl, 1914

Dercitus loricatus Lebwohl 1914: 84, pl. IX figs 21–60.
Pachastrella monilifera ; Maldonado 2002: 155 (not Schmidt 1868).

###### Material examined.

None.

###### Description

(from Lebwohl, 1914). Encrusting a lithistid (Discodermia) and partly overgrown by Pachastrella tenuilaminaris. Colour brownish yellow. Consistency compact in dry condition. Surface covered by dirt, serpulids, foraminifera and bryozoans, amidst two openings with diaphragms supposed to be oscules. Surface skeleton a thick crust of microrhabds and metasters. The interior is a mass of calthrops interspersed by metasters.

Spicules: calthrops, microrhabds, metasters.

Calthrops variable in number of cladi, often with bifid endings, but no real dichocalthrops, quite variable in size: 40–1250 × 5–165 µm.

Microrhabds ovoid, occasionally globular, rugose or microspined, 11.5–17.5 × 5.5–8.5 µm.

Metasters, microspined, 14–26 µm, with 4–18 rays of 5–20 µm length.

###### Habitat.

Deep water, 214 m.

###### Distribution.

Japan.

###### Remarks.

This is a dubious description as it was partly overgrown by a Pachastrella species, which might have contaminated the spicule complement (e.g. the microrhabds which are characteristic for Pachastrella). Since no sanidasters nor euasters were described, we exclude this material from our review as a pachastrellid of uncertain affinity. Maldonado (2002) assigned this to Pachastrella monilifera, but there is insufficient evidence for this.

##### Stoeba natalensis

Burton, 1926

Stoeba natalensis Burton 1926: 14.

###### Material examined.

None.

###### Description

(from Burton, 1926). Encrusting on Stelletta specimens. Dry condition. Skeleton made up by radially arranged dichotriaenes with rhabd (720 × 54 µm) approx. twice as long as the cladome (approx. 360 µm), making them long-shafted triaenes rather than dichocalthrops. Microscleres are called ataxasters ‘passing into tuberculate microrhabds’, 5–8 µm long.

###### Habitat.

Deeper water.

###### Distribution.

Natal coast, South Africa.

###### Remarks.

This is a dubious Dercitus (Stoeba) species and it needs redescription. Probably it should be assigned to an ancorinid genus.

### Family Calthropellidae

#### Genus Calthropella

Sollas, 1888

Calthropella Sollas 1888: 107.
Pachastrissa Lendenfeld 1903: 80.
Pachataxa de Laubenfels 1936: 179.
Corticellopsis Bergquist 1968: 62.

##### Emended definition:

Calthropellidae with calthrops or short-shafted triaenes and their dichotriaene modifications as megascleres, supplemented in some species with auxiliary thin oxeas. Microscleres euasters and in some members aster- or rhabd-derived microspined forms.

##### Type species:

Calthropella simplex Sollas, 1888.

Comments: Calthropella species are diverse in megasclere and microsclere composition reflected in several generic names currently in use or considered synonyms. We propose to reassign Calthropella species as here employed, to three subgenera, including Corticellopsis, formerly a synonym, but now revived to encompass those Calthropella species having only ‘normal’ euasters, Pachataxa, formerly a valid genus, to encompass species with deformed microspined polyangular microscleres (‘ataxasters’) along with normal oxyasters, and Calthropella s.s. for species having globular asters with characteristically tuberculated rays next to normal asters. Several new species of all three subgenera have been found in various parts of the world and will be described below.

The genus Pachastrissa is subsumed into the subgenus Calthropella (Calthropella) because the type species, Pachastrella geodioides (cf. below) differs only from the type of Calthropella, Calthropella simplex, in possessing an unstable complement of single oxeas, without structural position in the skeleton, often broken, and occasionally absent. Below, we maintain separate species Calthropella (Calthropella) simplex and Calthropella (Calthropella) geodioides, because we did not examine the type of the former, but Sollas’ description of it makes it clear that it differs from Calthropella (Calthropella) geodioides in two doubtful features, lack of dichocalthrops (rarely present in Calthropella (Calthropella) geodioides) and oxeas (unstable presence in Calthropella (Calthropella) geodioides, see below). Especially the shape of the globular asters in both is so similar that conspecificity is likely. Likewise Pachastrissa pathologica (Schmidt, 1868), and Pachastrissa inopinata (Pulitzer-Finali, 1983) are returned to Calthropella (Calthropella), close to Calthropella (Calthropella) geodioides.

The junior synonymy proposed for Pachastrissa as Calthropella does not extend to Pachastrissa hartmeyeri Uliczka (1929) and Pachastrissa nux (de Laubenfels, 1954) as Jasplakina, both of which fit better in the ancorinid genus Penares on account of the structural oxeas and the presence of ectosmal microxeas in these species.

The newly defined genus Calthropella is distinct from Dercitus s.l. in the possession of true euasters, the lack of sanidasters, the lack of large darkly pigmented cells with inclusions, and in the harder less organic texture of most species (except subgenus Corticellopsis). Calthrops are the dominant megasclere type, varying widely in size and shape (cladi 40–800 µm), with dichocalthrops so far known only from Calthropella (Calthropella) geodioides and its Indonesian ‘variety’. Asters are basically oxyasters, but they are often variable in shape with a strikingly high frequency of spheroxyasters with irregular shapes culminating in peculiar globular silica balls ornamented with tuberculated protrusions in some species. The subgenus Pachataxa has asymmetrical, deformed microspined siliceous microscleres which are derivatives of asters or microrhabds (which of these is unclear, so far). Differentiation in two aster types is usual. Like with several Dercitus species, oxea megascleres are reported in several descriptions, with widely different sizes and usually in broken condition. The oxeas may be foreign, but the persistent reports of their presence in some species indicate otherwise. In any case they do not have a structural position in the skeleton and are here considered ‘auxiliary’.

##### Key to the subgenera of Calthropella (see Fig. 23).

**Table:** 

1	Microscleres include only ‘normal’ asters (oxyasters and strongylasters)	Corticellopsis
–	Microscleres include next to ‘normal’ asters also highly silicified globular or irregular shapes	2
2	Microscleres include ‘ataxasters’ (irregular branched or ovoid microspined forms)	Pachataxa
–	Microscleres include regular tuberculated globular asters	Calthropella

**Figure 23.**
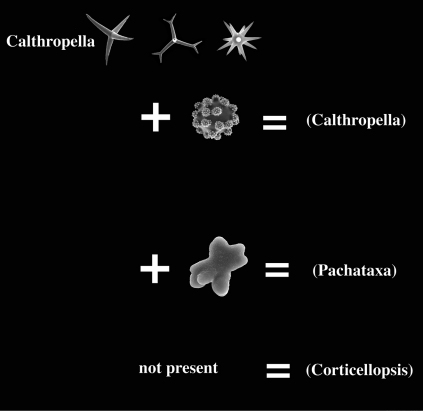
Key to the subgenera of Calthropella.

##### Subgenus Calthropella

Sollas, 1888

Calthropella Sollas 1888: 107.

###### Definition:

Calthropella with tuberculate spherasters.

###### Type species:

Calthropella simplex Sollas, 1888.

###### Key to the species of Calthropella (Calthropella).

**Table:** 

1	Dichocalthrops present	2
–	No dichocalthrops	3
2	Cladi of calthrops up to 800 µm	Calthropella (Calthropella) geodioides
–	Cladi of the calthrops only 210 µm	Calthropella (Calthropella) geodioides var. Ambon
3	Larger calthrops predominantly reduced to ‘tripods’ (three-claded megascleres); smaller microscleres deformed to form highly silicified spherical bodies spined all over	Calthropella (Calthropella) durissima
–	Larger calthrops variable, not predominantly ‘tripods’; microscleres recognizably spherasters and oxyasters	4
4	Calthrops extremely variable in shape, many with reduced number of cladi	5
–	Calthrops regular, but may have an extra cladus	6
5	Two categories of oxyasters; smallest oxyasters smooth	Calthropella (Calthropella) pathologica
–	Single oxyaster category, all microspined	Calthropella (Calthropella) inopinata
6	Oxyasters in two size categories	Calthropella (Calthropella) xavierae sp. n.
–	Oxyasters in a single size category	Calthropella (Calthropella) simplex

###### Calthropella (Calthropella) simplex

Sollas, 1888

Calthropella simplex Sollas 1888: 107, pl. X figs 13–14, 21–29; Topsent 1892: 42, pl. VII fig. 9.

####### Material examined.

None. Type material could not be found in the Natural History Museum (query in 2009).

####### Description

(from Sollas 1888 and Topsent 1892). Massive, with irregular upper surface, in places with tuberculate oscules; pale yellow to grey. Size up to 9×4.5×3.8 cm (Sollas’ type). Ectosomal cover of globular asters. Choanosome cavernous, but densely spiculated with a mass of calthrops.

Spicules: calthrops, globular spherasters and ‘normal’ oxyspherasters.

Calthrops, of widely different sizes, according to Sollas divisible into larger (cladi dimensions 800 × 90 µm) and smaller (150 × 20 µm).

Spheroxyasters, considered a young stage of the tuberculated asters by Sollas, but identified as a proper separate aster-type by Topsent; no size given by either authors but according to Topsent the same size as the truncated asters, so 12–24 µm in diameter.

Globular spherasters with tuberculated rays, variable, often irregular, in a wide size range, possibly divisible in two size categories, 24 µm and 12 µm.

####### Habitat.

Deep water, on gravel/sand bottom. Sollas provides no depth data, Topsent’s material came from 454 –861 m.

####### Distribution.

Cape Verde Islands (off Porto Praya, Santiago Island), Azores (38–39°N 30°W).

####### Remarks.

This species differs from the closely similar Calthropella (Calthropella) geodioides in the lack of dichocalthrops (but these are small and rare in Calthropella (Calthropella) geodioides) and oxeas (but these are an unstable complement not certainly proper in Calthropella (Calthropella) geodioides). Apart from Sollas (1888) and Topsent (1904) (who expressed doubts whether Calthropella simplex and Calthropella geodioides might be synonyms), no records of this species have been published. It is also closely similar to a new species from Indonesia, Calthropella (Calthropella) xavierae sp. n. (see below). Topsent (1892) distinguished a variety ‘*durissima*’ for specimens with globular spherasters becoming entirely irregularly rounded in some specimens; these specimens are here assigned to a separate species of Calthropella (Calthropella) (see below).

**Table 5.** Spicule data for Calthropella (Calthropella) species.

Author	calthrops cladi	oxeas	tuberculated sphaerasters	oxyasters	dicho- calthrops
Calthropella (Calthropella) simplex					
Sollas 1888 (Cape Verde Islands)	150–800×20–90	not recorded	12–24	yes	no
Calthropella (Calthropella) geodioides					
Carter 1876 (type) Sollas 1888 (Portugal)	700×85	736×10	25	not recorded	yes
Topsent 1904 (as Corticella, Azores)	yes, large variation	not recorded	20	12–15	yes
present paper (Azores)	102–705× 11–128	broken, few	7–28	13–18	yes
Calthropella (Calthropella) durissima					
present paper (Azores)	61–679× 4–96	800–1450 9–13	(1) 7–30 (2) 7–36	(1) 18–24 (2) 11–18	no
Calthropella pathologica					
Schmidt 1868 (Algeria) Topsent, 1938	550×75	long × 15	20–23	12–15	no
present paper MNHN DT 754	32–366× 5–72	2000×12	9–24	(1) 23–27 (2) 9–12	no
Vacelet 1969 (Marseille region)	60–800× 5–100	long × 12	15–22	12	no
Pouliquen 1972 (Marseille region)	40–600× 5–70	broken × 5–12	10–18	10–18	no
Voultsiadou and Vafidis 2004 (Aegean Sea)	50–495× 3.6–50	2000×15	7–17.6	7.2–17.6	no
Calthropella inopinata					
Pulitzer-Finali 1983 (Italy)	34–600	2000×15	5–22	8–17	no
Calthropella xavierae sp. n.					
present paper (Indonesia)	44–587× 5–101	not present	(1) 18–27 (2) 6–10	(1) 15–21 (2) 8–10	no
Calthropella simplex var. Topsent, 1897 (Ambon)					
Desqueyroux-Faúndez 1981	180–220	750×20	10–12	not recorded	yes

###### Calthropella (Calthropella) geodioides

(Carter, 1876)

Figs 24A–G

Pachastrella geodioides Carter 1876: 407, pl. XIV fig. 23.
Calthropella geodiides  (sic); Sollas 1888: 111.
Corticella geodioides ; Topsent 1904: 77, pl. IV fig. 14, pl. X fig. 12.
Calthropella geodioides ; Burton 1956: 142.
Pachastrissa geodioides ; Lendenfeld 1903: 80; Van Soest and Hooper 2002: 132, fig. 2D.

####### Material examined.

ZMA Por. 21666, EMEPC/G3–D03A–Ma012, Azores, Terceira Island, 38.4265°N; 26.8206°W, 1201 m, coll. J. Xavier, 18 May 2007.

Specimens provided by Dr Joana Xavier, as yet unregistered: EMEPC/G3–D01–Ma005, Azores, São Jorge Island, 38.48°N; 27.798°W, 1222 m, 17 May 2007; EMEPC/G3–D02–Ma006, Azores, Terceira Island, 38.5530°N; 26.7083°W, 744 m, 18 May 2007; EMEPC/G3–D33A–Ma011a, S of Azores, Cruiser Seamount, 32.2570°N; 27.553°W, 643 m, 4 June 2007; EMEPC/G3/08.30, S of Azores, Atlantis Seamount, 33.916°N; 30.171°W, 1132 m, 30 October 2008.

####### Description.

**Figure 24.**
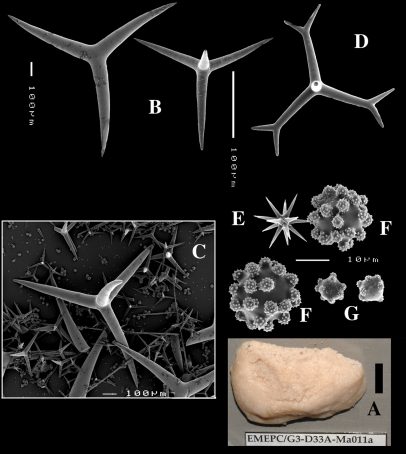
Calthropella (Calthropella) geodioides, ZMA Por. 21666, from Azores, Terceira Island, **A** habit (scale bar 1 cm) **B** calthrops **C** overview of spicules, including broken oxeas **D** dichocalthrops **E** oxyaster **F** large tuberculated spherasters **G** small tuberculated spherasters.

Flat mounds (Fig. 24A) and thick crusts, rounded off at the edges. Size up to 5×3.5×1 cm. Surface generally smooth-looking and lacking obvious apertures. Rough to the touch, hard but crumbly. Colour white or cream (alcohol).

Skeleton: with a dense crust of microscleres carried by triaene megascleres, with scattered bundles of oxeas, which appear mostly broken and are sometimes entirely absent.

Spicules: calthrops, dichocalthrops, (broken) oxeas, tuberculated spherasters, oxyasters.

Calthrops (Fig. 24B–C), with common triactine forms, in a wide size range, cladi 102–*350.9*–705 × 11–*51.5*–128 µm, cladomes 150–*471.4*–1050 µm, possibly divisible in two categories (cladi lengths 102–180 and 434–705 µm).

Dichocalthrops (Fig. 24D) , few (absent in some specimens) and fairly small: protocladi 75–92 × 12 µm, deuterocladi 28–31 µm, rhabdome 92–120 µm, cladome 128–183 µm.

Oxeas (visible in Fig. 24B) invariably broken, at least 500 µm in length and 5 µm diameter. Absent in some specimens (Sollas gives oxea size 736 × 9.3 µm).

Oxyasters (Fig. 24E) with conical rays, perhaps to be considered oxyspherasters, not common, 13–18 µm.

Tuberculated spherasters (Fig. 24F–G), quite variable, in some specimens entirely smooth and of irregular oval shape, usually in a large size range 7–28 µm, possibly divisible in two overlapping size classes (7–12 and 23–28 µm).

####### Habitat.

On rocks in deep water, 599–1222 m.

####### Distribution.

Off Cabo São Vicente, Portugal; Azores. Burton (1956) lists the species for Madeira, but the basis for that is not clear.

####### Remarks.

Topsent (1904) and Pulitzer-Finali (1983) both point out the great similarity of Calthropella simplex and Calthropella geodioides. Points of difference apparently are the absence of dichocalthrops and oxeas in Calthropella simplex. In our present series of samples we could not find any oxeas in samples Ma012 and Ma005 (see above) and in the other samples they were invariably single, not forming bundles or radiating towards the surface and appeared to be broken generally in all sections and spicule mounts. Possibly this was artefactual. Dichocalthrops were rare to extremely rare in all studied samples. Dichocalthrops that were encountered were all in the small size class of triaene megascleres (less than 200 µm cladome size) and often were not ‘complete’, i.e. only one or two of the cladi were forked.

We believe that the two species *simplex* and *geodioides* could very well be the same species and could eventually be merged, but we will await further studies including molecular sequencing.

Topsent’s (1897) variety of Calthropella geodioides from Ambon (Indonesia) is not close to the present species. It shares the presence of oxeas and dichocalthrops with Calthropella geodioides but calthrops are much smaller (200 µm) and thick–centred euasters are only 10–12 µm. It is redescribed in Desqueyroux-Faúndez (1981) but remains ill-known (see below).

###### Calthropella (Calthropella) durissima

Topsent, 1892

Figs 25A–F

Calthropella simplex var. durissima Topsent 1892: 43, pl. V fig. 14, Pl. VIII fig. 9.

####### Material examined.

Specimen provided by Dr. Joana Xavier, as yet unregistered: EMEPC/G3/08.30, S of Azores, Atlantis Seamount, 33.916°N; 30.171°W, 1132 m, 30 October 2008.

####### Holotype.

Not examined. Dr. P. Cárdenas (*in litteris*) reports the existence of a slide, MNHN DT 883.

####### Description.

**Figure 25.**
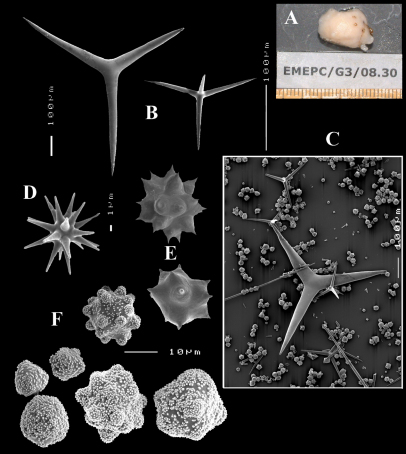
Calthropella (Calthropella) durissima, specimen EMEPC/G3/08.30, from S of Azores, Atlantis Seamount **A** habit **B** ‘tripod’ and calthrops **C** overview of spicules **D** oxyaster **E** smooth spheroxyasters **F** tuberculated spheraster and various derived slica bodies.

White, massive lump (Fig. 25A), encrusting volcanic rock, with optically smooth surface and hard consistency. Size approx. 2×1.5×1 cm.

Skeleton: largely confused interiorly, with a densely crowded outer layer of microscleres.

Spicules: calthrops, two types of oxyasters, tuberculated asters, lumpy asters; some broken oxeas.

Calthrops (Fig. 25B–C) variable in size and shape, possibly divisible in two broadly overlapping size categories; the largest are often ‘tripods’ (three–claded calthrops) (Fig. 25B–C), among the smaller are five–claded modifications with one or more cladi shorter or vestigial, cladi 61–*327.3*–679 × 4–*44.2*–96 µm (cladomes 98–955 µm), smaller with cladi in the range 61–198 µm are usually normal calthrops, larger with cladi in the range 297–697 µm are predominantly tripods.

Oxeas (Fig. 25B), few in number and invariably broken, smallest / largest fragment 800–1450 × 9–13 µm.

Oxyasters, occurring in two distinct types: small oxyasters (Fig. 25D) with small center and long conical but thin rays, diameter 11–*13.7*–18 µm; larger spherasters (Fig. 25E) with conical smooth rays ending in a single spine, relatively rare, diameter 18–24 µm (N=5).

Tuberculated asters (Fig. 25F) with short rays (almost entirely taken up by the tubercles), rare and grading into lumpy asters, diameter 7–30 µm (N=4).

Lumpy, irregular aster-derived silica bodies (Fig. 25F), probably derived from tuberculated asters, but most become entirely irregularly rounded: 7–*21.5*–36 µm. These asters were named ‘spherochiasters’ by Topsent (1904).

####### Habitat.

Deep water, 450–1132 m.

####### Distribution.

W of Flores and seamounts S of the Azores.

####### Remarks.

Topsent’s variety *durissima* (type specimen MNHN DT 833 from deep water off the Azores) is here raised to species rank because of the peculiar modification of the tuberculated asters into small irregular silica-balls, which are also larger than those of Calthropella (Calthropella) simplex and Calthropella (Calthropella) geodioides. Topsent’s type material was more extensive (largest specimen had half the size of a fist) and the surface more elaborate (‘rugueux’, oscules sometimes on a papilla). In 1904, Topsent retracted his variety *durissima* as he had become convinced that the lumpy asters were derived from the tuberculated asters and probably subject to considerable variation. Having examined the variation of these asters, we disagree with the retraction of *durissima* and Topsent’s assignment of it to the variability of Calthropella (Calthropella) geodioides. The predominance of three-claded calthrops and the absence of dichocalthrops combined with the presence of the lumpy entirely spined ‘spherochiasters’ is sufficient evidence for specific distinction.

###### Calthropella (Calthropella) pathologica

(Schmidt, 1868)

Figs 26A–F

Stelletta pathologica Schmidt 1868: 19, pl. III figs 3–4.
Calthropella pathologica ; Topsent 1938: 24; Vacelet 1969: 166, fig. 3; Pouliquen 1972: 746, pl. 7 fig. 3; Maldonado 1992: table 1.
Pachastrissa pathologica ; Voultsiadou and Vafidis 2004: 593.

####### Material examined.

Lectotype (designation herein), MNHN DT 753, Coast of Algiers, Expédition Scientifique de l’Algérie, nr. 66, 1842. Paralectotype MNHN DT 754, from same locality.

####### Redescription

**Figure 26.**
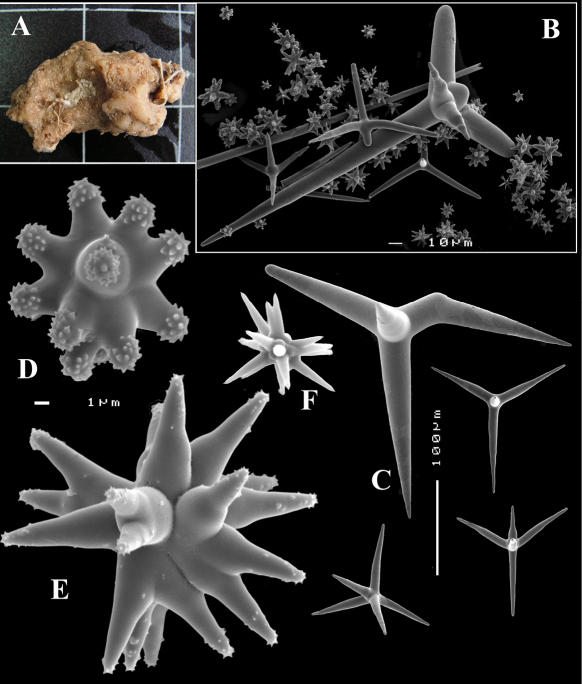
Calthropella (Calthropella) pathologica, lectotype MNHN DT 753 and paralectotype MNHN DT 754, from Algiers, **A** habit of lectotype (grid is 1 cm) **B–F** SEM images made from the paralectotype, **B** overview of spicules **C** various calthrops including a pentactinal modification **D** tuberculated spheraster **E** large irregular oxyspheraster **F** small oxyaster.

(partly from Topsent, 1938). Two specimens are present in the Schmidt collection of the Muséum National d’Histoire Naturelle, Paris, the largest (MNHN DT 753) of which is 4.5 cm in widest size and 2–2.5 cm high, here chosen as the lectotype. The smaller specimen (MNHN DT 754), here designated paralectotype, size 1×3 cm (now apparently reduced to 1×1.5 cm) was examined by us and we made SEM photos of the spicules. Both specimens have a rough surface (Fig. 26A). According to Vaclelet (1969) living specimens are white, but the type specimens we examined were yellowish in alcohol.

Spicules: Calthrops, tuberculated spherasters, oxyasters.

Calthrops (Figs 26B–C): According to Topsent the skeleton includes calthrops with cladi up to 550 × 75 µm (but widely different sizes are present, including ‘microcalthropes’). We measured a size range of the cladi of 32–366 × 5–72 µm (cladomes 48–480 µm) but no clear separation in smaller and larger size categories was apparent. The shapes of the calthrops was very variable with many ‘short-shafted triaenes’, mesotriaene modifications, curved and stunted cladi. No dichocalthrops are reported from this species.

The megasclere complement also comprised thin oxeas of 15 µm thickness (Fig. 26B). Cross sections of the type specimens examined by P. Cárdenas show scattered bundles of the thin oxeas running vertically to the surface, but their length still is difficult to determine. We are indebted to P. Cárdenas for this information. The largest unbroken piece we found in preparations of DT 754 was 2000 × 12 µm, which is in accordance with findings of Voultsiadou and Vafidis (2004).

Asters (Figs 26D–F) occurred in three distinct categories: thick-centred with spined-tuberculated rays, asters with thick pointed rays lighly spined, and small smooth oxyasters.

Lightly spined oxyasters (Fig. 26E) with swollen pointed rays, often with bifid rays, not very common, diameter 23–*24.9*–27 µm.

Small, smooth oxyasters (Fig. 26F), often slightly irregular in ray length, diameter 9–*10.2*–12 µm.

Tuberculated asters with ornamented rays (Fig. 26D); compared to Calthropella (Calthropella) geodioides the rays were relatively long; sizes highly variable, but overlapping without clear separation in smaller and larger asters, diameter 9–*18.4*–24 µm

####### Habitat.

Deep water, and in caves in more shallow water, depth occurrence at least 13–250 m. Vacelet (1969) reported that his specimen was insinuating in calcareous algae.

####### Distribution.

Off the coast of Algeria, no further data; Cassidaigne, Marseille region; Sicily-Tunis region; Alboran Sea, 39°N –3°W; Rhodos, Aegean Sea, 36°N; 28°E.

####### Remarks.

Topsent (1938) remarked that the specimens are similar in most aspects to Calthropella geodioides but differ in the tendency of the calthrops to develop extra cladi. In addition the tuberculated asters of that species have shorter rays (almost entirely consisting of tubercles). Oxeas are stated in Voultsiadou and Vafidis (2004) to be all broken, whereas Vacelet (1969) notes ‘wide axial canals’, adding to the conclusion that they are not of structural significance. Nevertheless, the bundles reported by P. Cárdenas (*in litteris*) appear to indicate these oxeas are proper to the sponge. Possibly, they are a remnant of ancestral radiating oxeas.

###### Calthropella (Calthropella) inopinata

Pulitzer-Finali, 1983

Calthropella inopinata Pulitzer-Finali 1983: 464, figs 9–10.

####### Material examined.

None.

####### Holotype.

MSNG 47158, off Camogli, N Italy.

####### Description

(from Pulitzer-Finali 1983). Irregularly lobate, stony hard sponges, size up to 15×12×2 cm. Surface ‘even’, no apparent oscules. Light yellow in dry condition.

Spicules: calthrops, oxeas, strongylasters to spherasters, oxyasters.

Calthrops, extremely variable in shape, with many reductions and malformations, size variation also considerable, 34–600 µm (no thickness given).

Oxeas, with extremely elongated, flexuous points, all broken in the slide, but at least 2000 × 15 µm.

Oxyasters, without centre, with two to six microspined rays, 8–17 µm.

Strongylasters to spherasters, variable in shape, irregular with tuberculated rays or lobate, 5–22 µm.

####### Habitat.

Trawled from 60 m depth.

####### Distribution.

Known only from the type locality off the coast of Genoa, 44°N; 9°E.

####### Remarks.

This species is similar to Calthropella pathologica in most aspects. Oxeas are stated to be all broken, so chances are they are not proper or only auxiliary. Nevertheless their great length and peculiar endings are the main reason to distinguish this species as separate.

Pulitzer (l.c.) suggests that Calthropella geodioides, Calthropella simplex, Calthropella pathologica and his new species Calthropella inopinata could be all members of the same species. For us, this suggestion has merit, at least as far as a possible synonymy of Calthropella pathologica – Calthropella inopinata and Calthropella simplex – Calthropella geodioides is concerned. A further North Atlantic species, Calthropella (Calthropella) durissima differs clearly in having peculiar silicified microscleres derived from the tuberculated asters.

###### Calthropella (Calthropella) xavierae sp. n.

urn:lsid:zoobank.org:act:CFED089B-4226-4FEA-84C2-71481A291F48

Figs 27A–G 

####### Material examined.

Holotype ZMA Por. 11376, Indonesia, E of Komodo, 8.4867°S; 119.6167°E, depth 138 m, dredge, coll. R.W.M. Van Soest, Snellius II Exped. Stat. 095, 19 September 1984.

####### Description.

**Figure 27.**
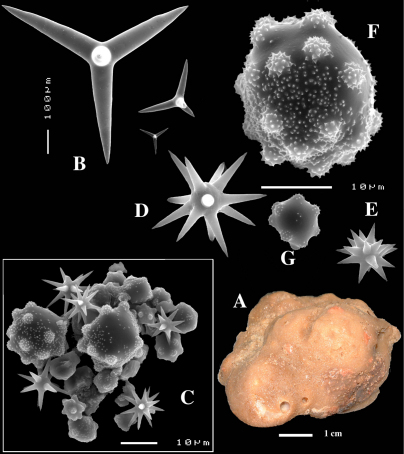
Calthropella (Calthropella) xavierae sp. n., holotype ZMA Por. 11376, from Indonesia, E of Komodo, **A** habit **B** calthrops **C** overview of asters **D** large oxyaster **E** small oxyaster **F** large tuberculated speraster **G** small tuberculated spheraster.

Lobate mass (Fig. 27A), size 6×5×5cm, microhispid surface, rough to the touch. Colour pinkish brown. Two oscule type openings are located on the rounded upper surface, 3–4 mm in diameter. Consistency hard.

Skeleton: a dense crust of asterose microscleres at the surface covers a dense mass of calthrops. Asters are also crowded in the choanosome. The skeleton is densely confused and there are few canals or cavities; some broken monaxone spicules were present.

Spicules: calthrops, globular tuberculate asters, oxyspherasters, oxeas?.

Calthrops (Fig. 27B) in an extremely wide range of sizes and shapes, cladi 44–*238.2*–587 × 5–*40.8*–101 µm, cladome 62–*344.3*–820 µm.

Broken monaxonic spicules (oxeas?) measured up to 700 × 10 µm.

Oxyasters (Fig. 27C), with a discrete rounded center and rays often ill developed or partly missing, range from 8 to 21, possibly in two size categories, the larger (Fig. 27D) with fewer rays, 15–*16.9*–21 (8 rays) and smaller (Fig. 27E) with more numerous rays, 8–*9.7*–10 µm (12–14 rays), but some overlap is present.

Globular tuberculated spherasters (Fig. 27C) or ‘silica balls’, appearing to be derived from the normal euasters by heavy silicification, in a wide size range, 6–27 µm, possibly in two size categories, larger (Fig. 27F) 18–*21.3*–27 µm and smaller (Fig. 27G) 6–*8.3*–10 µm.

####### Etymology.

The name of the new species acknowledges all the help we received from Joana B.R.T. Xavier during her stay in Amsterdam, and the generous donation of several sponge fragments important for this study.

####### Habitat.

Dredged from hard bottom at 138 m.

####### Distribution.

Known only from the type locality, E of the island of Komodo, Nusa Tengara (southern island chain bordering the Banda Sea).

####### Remarks.

This is a clear Calthropella (Calthropella) resembling the type species Calthropella (Calthropella) simplex in all but the finer details of spicule sizes and shapes. The habitus is more elaborately globular and has pinkish brown colour (in alcohol) in contrast to more flattened pale yellow–grey Calthropella (Calthropella) simplex; there appear to be two distinct size categories of oxyspherasters in the new species.

No matching descriptions are found in the Indo–West Pacific region, but Topsent (1897) reported the occurrence of Calthropella geodioides var. from Ambon Bay, Indonesia (see also below based on a redescription in Desqueyroux-Faúndez 1981) which shows a few similarities. It differs from our new species at least by the possession of dichocalthrops, apparently proper oxeas and white colour.

###### Calthropella (Calthropella) geodioides

var. Ambon

Calthropella geodioides  var. Topsent 1897: 438.
Calthropella geodioides ; Desqueyroux-Faúndez 1981: 730, Figs 13–15, 106.

####### Material examined.

None. According to Desqueyroux-Faúndez (1981) there are several specimens in MHNG (nr. C12/12).

####### Description

(from Topsent 1897 and Desqueyroux-Faúndez 1981). This is described as a small white sponge, similar to the North Atlantic Calthropella geodioides, but differing by possessing calthrops with a fifth cladus, and by rare microscleres. Calthrops are much smaller than in the North Atlantic material, with cladi length 180–210 µm, whereas the dichocalthrops are much larger than those of Calthropella geodioides proper, being of similar size as the calthrops. There are thick-centred tuberculated asters with truncated rays, 10–12 µm. Further megascleres are oxeas of 750 × 20 µm (not pictured by Desqueyroux-Faúndez).

####### Habitat.

Shallow reefs.

####### Distribution.

Ambon Bay, Indonesia.

####### Remarks.

It is likely that the Ambon material belongs to an undescribed species, as the length of the cladi of the calthrops and the diameter of the tuberculated asters are much smaller than in the type of Calthropella geodioides, and ‘normal’ oxyasters are apparently not present. In addition, the localities and habitats are widely separated (deep sea off Portugal vs. Ambon Bay).

##### Subgenus Pachataxa

de Laubenfels, 1936

Pachataxa de Laubenfels 1936: 179.

###### Definition:

Calthropella with ataxasters.

###### Type species:

Pachastrella lithistina Schmidt, 1880 (by original designation).

###### Key to the species of Pachataxa.

**Table:** 

1	Oxyasters up to 24 µm, ataxasters predominantly pear-shaped	Calthropella (Pachataxa) pyrifera sp. n.
–	Oxyasters less than 12 µm, ataxasters irregularly branched	2
2	Ataxasters up to 48 × 34 µm, sponge plate-shaped	Calthropella (Pachataxa) lithistina
–	Ataxasters up to 42 × 16 µm, sponge lobate	Calthropella (Pachataxa) enigmatica

###### Calthropella (Pachataxa) lithistina

(Schmidt, 1880)

Figs 28A–D 29A–E

Pachastrella lithistina Schmidt 1880: 68, pl. 9 fig. 3; Topsent 1923: 6, fig. 1.
Pachataxa lithistina ; de Laubenfels 1936: 179; Van Soest and Hooper 2002: 133, fig. 2E.

####### Material examined.

Holotype MCZ 6384, Blake Exped. 1878–79, Grenada, 160 m. Further type material: schizoholotype fragment MZUS P0095 (not examined) and two type slides in ZMB, nr. 6870 (not examined).

####### Description.

**Figure 28.**
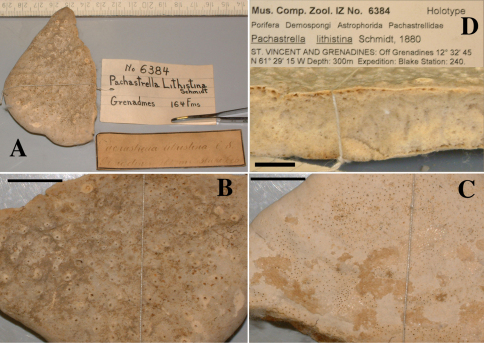
Calthropella (Pachataxa) lithistina, holotype MCZ 6384, from Grenada, **A** overview of type **B** detail of oscular surface **C** detail of poral surface **D** cross section of choanosome.

Thick plate (Fig. 28), size 9×6 cm, thickness 2 cm. Surface different on both sides, oscular side (Fig. 28B) with irregularly scattered oscules of 1–2 mm diameter each elevated on small hillocks; the poral side (Fig. 28C) with numerous small openings irregularly arranged in groups separated by areas without such openings. Edges of the plate smooth, without pores or oscules. Colour of the dried specimen pale yellow with a pinkish brown tinge. Consistency hard, slightly crumbly (dry condition). In cross section (Fig. 28D), there is a dense interior yellow-white mass, riddled with thin canals; at the periphery there are subdermal spaces underneath a 1 mm thick crust.

Skeleton: a confused mass of calthrops with at the periphery a dense mass of ataxasters, which are also strewn in the interior.

Spicules: calthrops, spheroxyasters, ataxasters.

**Figure 29.**
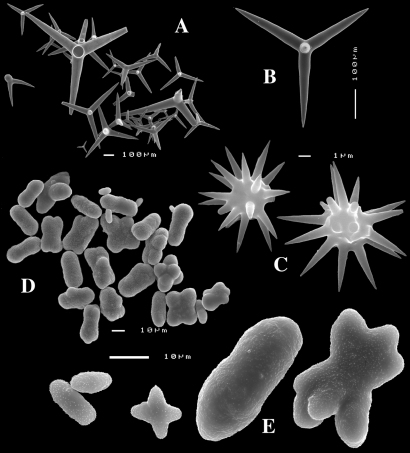
Calthropella (Pachataxa) lithistina, holotype MCZ 6384, **A** overview of calthrops **B** detail of calthrops **C** oxyasters **D** overview of ataxasters **E** various ataxasters

Calthrops (Fig. 29A–B), highly variable in size, cladi conical and straight, occasionally the fourth cladus is lacking or underdeveloped, rarely one of the cladi is longer, 78–*315.5*–705 × 14–*46.1*–94 µm, cladome 144–*460.2*–990 µm.

Spheroxyasters (Fig. 29C), with thick center and smooth conical rays of unequal length, some appear undeveloped, many are broken; diameter (including the rays) 7–*9.7*–12 µm.

Ataxasters, of many different shapes and sizes (Fig. 29D–E) from simple elongate-ovate rhabds to complicated forms looking as if two or more rhabds are fused, surface uniformly microspined; size (length of longest axis × length of shortest axis) 9–*28.7*–48 × 4–*18.1*–34 µm.

####### Habitat.

Deep water, 160 m.

####### Distribution.

Only known from the type locality, off Grenada.

####### Remarks.

The type material was assigned by Van Soest and Hooper (2002) to a lectotype in the Strasbourg Museum and a paralectotype in the Museum of Comparative Zoology, Cambridge, USA, but it is virtually certain that both are fragments of a single specimen. Thus, both the alleged lectotype and paralectotype are now assigned to the same holotype as schizoholotypes. Calthropella (Pachataxa) lithistina differs from both other Pachataxa species in the shape (large plate with different oscular and poral surfaces) and in the large variations in ataxaster shapes and sizes.

###### Calthropella (Pachataxa) enigmatica

Lévi & Lévi, 1983

Figs 30A–F

Pachataxa enigmatica Lévi and Lévi 1983: 151, fig. 29.

####### Material examined.

Holotype MNHN DCL 2805; paratypes DCL 2895 (4 small sponges), both from New Caledonia, 22.8°S; 167.15°E, depth 355–360 m.

####### Description.

**Figure 30.**
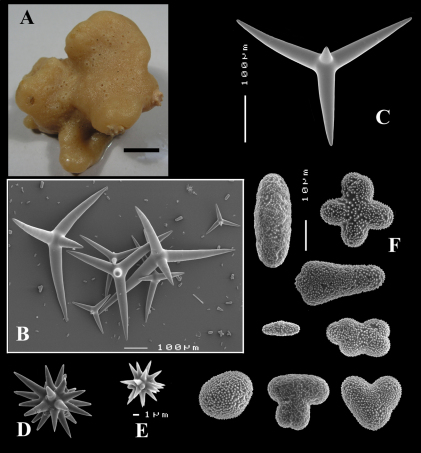
Calthropella (Pachataxa) enigmatica, paratype MNHN DCL 2895 from New Caledonia, **A** habit (photo P. Cárdenas, scale bar 1 cm) **B** overview of spicules **C** calthrops **D** larger oxyaster **E** smaller oxyaster **F** various ataxasters.

Massive with rounded flattened lobate outgrowths (Fig. 30A). Size of holotype 3.5×3.5×1.2 cm, paratypes slightly smaller to about 1 cm3. Brownish beige in alcohol. Small slightly raised oscules of less than 1 mm diameter are scattered in vague groups on the upper surface. Consistency firm to hard, crumbly.

Skeleton: confused, with a crust of microscleres carried by the triaene megascleres. A few broken oxea-like spicules were present in the slides made from the largest paratype.

Spicules (Fig. 30B): Calthrops, spheroxyasters, ataxasters.

Calthrops (Fig. 30B–C), occasionally with five-claded mesotriaene-like forms (called ‘centrotriaenes’ by Lévi and Lévi), cladi 70–*215.4*–450 × 13–*30.6*–54 µm, cladomes 120–*311*–510 µm.

Spheroxyasters (Figs 30D–E), called ‘chiasters’ by Lévi and Lévi, but they do not have blunt rays), small, 4–*8.7*–12 µm diameter (but not as small as 4–5 µm as Lévi and Lévi stated).

Ataxasters (Fig. 30F) are microspined, basically ovoid microrhabds, with many forms branched or irregular (shapes may be termed centrotylote rhabds, rods with side branch(es), round balls, cross-shaped, or rarely polyangular/aster-like), size ranging from 9 × 4 to 42 × 16 µm.

####### Habitat.

Deep water, 355 m.

####### Distribution.

Known only from off New Caledonia.

####### Remarks.

The New Caledonian species differs from the Caribbean Calthropella (Pachataxa) lithistina in overall spicule size and particularly in the small size of the oxyasters; that species is much larger and has a plate-shape. Calthropella (Pachataxa) pyrifera sp. n. (see below) differs likewise in having larger oxyasters up to 24 µm diameter and asymmetrical pear-shaped ataxasters.

###### Calthropella (Pachataxa) pyrifera sp. n.

urn:lsid:zoobank.org:act:F2A5D208-1076-45F4-95EE-EA57D4C2C028

Figs 31A–E 

####### Material examined.

Holotype HBOI nr. 12–XI–86–1–14, with fragment (schizoholotype) ZMA Por. 07726, Ecuador, Galapagos Archipelago, NE coast of Santa Cruz, Rocas Gordon, 0.546°S; 90.116°W, depth 506 m, coll. K. Rinehart, Johnson SeaLink submersible, 12 November 1996, don. S.A. Pomponi & M.C. Diaz.

####### Description.

**Figure 31.**
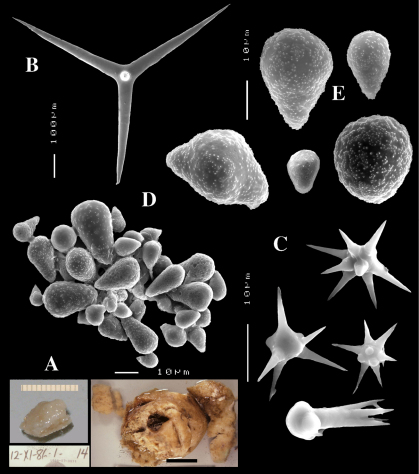
Calthropella (Pachataxa) pyrifera sp. n., from Ecuador, Galapagos Archipelago, NE coast of Santa Cruz, **A** habit, left of the schizoholotype ZMA Por. 07726, right of the holotype HBOI nr. 12–XI–86–1–14 (scale bar 1 cm) **B** calthrops **C** various oxyasters **D** group of ataxasters **E** various ataxasters.

Type material available to us has a flattened (Fig. 31A left), rounded shape, size 1×1×0.2 cm. It is a fragment of a larger holotype specimen (3.5×3×2.5 cm) that is massively rounded and may have a large central oscule (Fig. 31A right). Colour very pale green or dirty white.

Skeleton: an irregular mass of large triaenes covered at the periphery by a thick layer of ataxasters / microrhabds, which are also strewn in the interior. Loose oxeas of widely different sizes present in moderate quantities, considered foreign.

Spicules: calthrops, spheroxyasters, ataxasters.

Calthrops (Fig. 31B) variable in size and thickness, mostly with straight cladi, often one cladus slightly shorter, occasionally curved at the ends; cladi 133–*412.3*–708 × 15–*37.4*–71 µm, cladome 233–*626.8*–1180 µm.

Spheroxyasters (Fig. 31C), thick-centred with smooth conical rays, many of which appear underdeveloped causing an irregular aspect; extreme cases are the medusa-like forms such as pictured at lower right in Fig 31C, showing a deformed aster with rays present only at one side; diameter (including rays) 15–*18.1*–24 µm (centre approx. 10 µm diameter).

Ataxasters (Figs 31D–E), typically pyriform (pointed one-sidely), occasionally ovate or rounded, microspined all over, but spines tend to be grouped; no branching shapes were found, making the term ‘ataxaster’ inappropriate for these spicules, but their homology to the ataxasters of Pachataxa lithistina is nevertheless obvious; size 7–*20.5*–31 µm, measured along the longest axis.

####### Etymology.

From the Greek pyros = pear and fero= carry or bear, referring to the possession of the pear-shaped microscleres.

####### Habitat.

Deep water, 506 m.

####### Distribution.

NE of Santa Cruz Island, Galapagos, East Pacific.

####### Remarks.

The species is assigned to Calthropella (Pachataxa) on the basis of its spiculation. Size and shape of the calthrops is similar to Calthropella (Pachataxa) lithistina, but these differ clearly in the diameter of the spheroxyasters (those of Calthropella (Pachataxa) pyrifera sp. n. are twice as large and these are also twice as large as those of Pachataxa enigmatica, see above). Especially the shape of the ataxasters renders this species distinct from the other two Calthropella (Pachataxa).

##### Subgenus Corticellopsis

Bergquist, 1968

Corticella Sollas 1888: 281 (preoccupied by CorticellaEhrenberg 1872, Protoctista).

###### Definition:

Calthropella with ‘normal’ euasters (oxyasters and/or strongylasters).

###### Type species:

Corticium stelligerum Schmidt, 1868 (by subsequent designation herein).

###### Key to the species of Corticellopsis.

**Table:** 

1	Calthrops cladi small and thin, less than 195 × 10 µm	Calthropella (Corticellopsis) spec. Seychelles
–	Larger and thicker calthrops	2
2	Oxyasters up to 70 µm diameter	Calthropella (Corticellopsis) novaezealandiae
–	Oxyasters only up to 50 µm diameter	3
3	Auxiliary oxea megascleres present	Calthropella (Corticellopsis) recondita
–	No oxeas	Calthropella (Corticellopsis) stelligera

###### Calthropella (Corticellopsis) stelligera

(Schmidt, 1868)

Figs 32A–C

Corticium stelligerum Schmidt 1868: 25, pl. III fig. 6; Desqueyroux-Faúndez and Stone 1992: 10, pl. I figs 4–6.
Corticella stelligera ; Sollas 1888: 281; Lendenfeld 1894: 18, pl. II fig. 11, pl. III fig. 49; Topsent 1895: 339, pl. XXII fig. 1.
Corticellopsis stelligera ; Bergquist 1968: 62.
Calthropella stelligera ; Cruz and Bacallado 1982: 81; Cruz 2002: 89; Voultsiadou and Vafidis 2004: 593.

####### Material examined.

Type fragment of LMJG 15352 from Sebenico, Northern Adriatic (slide in ZMA). There are further fragments, not examined, BMNH 1867.7.26.14, 45, 104, BMNH 1910.1.1.863, MZUS P0005, and slides BMNH 1868.3.2.5 and ZMB 6563).

####### Description.

From Schmidt (1868) and Topsent (1895), based on a fragment presented to the British Museum (Natural History), BMNH 1910.1.1.863. Encrusting on corals and bridging crevices between coral branches, thickness about 5 mm. Colour white (in dry condition), yellowish inside. Desqueyroux-Faúndez and Stone (1992) picture the Schmidt specimen of which a fragment was studied from Sebenico as a massively encrusting sponge of 4×4 cm, encrusted and riddled with shells. According to Sollas (1888) it has a thickness of 5 mm. Cruz (2002) reports pale yellow or greyish live colour. The World Porifera Database (van Soest et al. 2008) has an in situ picture made by Dr B. Picton from a locality just off the coast of Marseille showing a pale yellow colour. No apparent oscules.

Skeleton: densely spiculous at the surface, organic with few spicules in the interior. No definite skeletal structure.

Spicules: calthrops, euasters.

**Figure 32.**
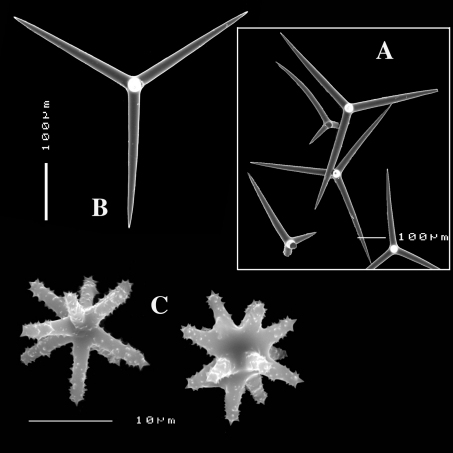
Calthropella (Corticellopsis) stelligera, one of the types, LMJG 15352 from Sebenico, Northern Adriatic, **A** overview of megascleres **B** calthrops **C** cortical strongylasters (oxyasters were not present in the studied fragment).

Calthrops (Fig. 32B) or short-shafted triaenes (Fig. 32A), variable in size, cladi 142–*288.2*–356 × 16–*25.6*–33 µm (cladomes 212–*436.0*–570 µm), Topsent gives cladus lengths 130–400 × 12–30 µm.

Microscleres euasters in two categories (although the LMJG fragment available to us only contained a single one: strongylasters 16–*20.2*–22 µm in diameter (Fig. 32C) with microspined rays in the studied fragment). Schmidt (1868) gives 20 µm as size of the strongylasters, whereas the oxyasters were measured by him as 50 µm. Topsent gives only ray lengths, 30 µm for the oxyasters (which could conform to 50 µm in overall diameter), and 4–7 µm for the ‘chiasters’ (which seems a bit too short for an overall 20 µm diameter mentioned by Schmidt). Presumably the various type specimens have considerable size variation in the asters.

####### Habitat.

Fairly shallow water, from intertidal caves to 20 m.

####### Distribution.

Adriatic; Marseille; Canary Islands; NW Aegean Sea, 39°N; 25°E (Algeria is given by previous authors, but this is probably based on a misunderstanding of Schmidt’s text).

####### Remarks.

The species is the type of the preoccupied genus Corticella Sollas (1888) replaced by Corticellopsis Bergquist, 1968 (see Van Soest and Hooper 2002 for details). It is apparently quite rare as there are only a few records from the Mediterranean (Northern Adriatic, Greece, and unconfirmed from Marseille). Schmidt (l.c.) mentions Algeria in his text, but very probably refers to another Corticium species (either Calthropella candelabrum or Calthropella plicatum = Dercitus plicatus). The species has been reported recently from the Canary Islands (Cruz and Bacallado 1982 and Cruz 2002) as pale yellow or grey masses with a skeleton of calthrops with cladi of 60–320 µm, irregular chiasters (strongylasters) of 8–23 µm diameter, drawn with smooth rays, five–rayed oxyasters also drawn with smooth rays of 16–40 µm. Voultsiadou and Vafidis (2004) give calthrops cladi of 120–240 × 20 µm, ‘chiasters’ with 5–8 µm ray lengths, and six–rayed oxyasters of 20–28 µm ray length. They also mention the presence of considerable amounts of oxeas, but these were assumed to be foreign. 	The various measurements of either ray length or overall diameter make it difficult to monitor the size variation of the asters. This species needs to be revised further, based on examination of all extant specimens.

###### Calthropella (Corticellopsis) recondita

Pulitzer-Finali, 1972

Calthropella recondita Pulitzer-Finali 1972: 342, fig. 3.

####### Material examined.

None. Apparently the material that is left consists only of slides, of which the holotype is in the Natural History Museum, London (BMNH 1971.7.23.1b).

####### Description

(from Pulitzer-Finali 1972). Thin encrustations on coral holdfasts, thickness a few mm. Consistency fleshy, compact. Colour white.

Spicules: calthrops, oxeas, strongylasters (called ‘chiasters’), oxyasters.

Calthrops in a large size variation and many showing abnormal or assymmetrical cladi (the latter distinguished as a separate category by Pulitzer), cladi 85–220 × 7–17 µm.

Oxeas straight or curved, in a large size range, 100–1200 × 7.5 µm.

Strongylasters, 8–12 rays, variable, 9–16 µm.

Oxyasters, six thin rays, 40–50 µm.

####### Habitat.

Collected at 12 m.

####### Distribution.

Mediterranean (Naples region).

####### Remarks.

The oxeas are explicitly stated as part of the spicule complement, but were not represented in the spicule drawings. It remains doubtful whether they are proper. Pulitzer-Finali (1972) admits that his species is similar in most aspects to Calthropella (Corticella) stelligera. By its possession of oxeas, assuming they are proper, this species would appear to be valid, but the possibility that it is Calthropella (Corticella) stelligera cannot be excluded.

###### Calthropella (Corticellopsis) novaezealandiae

(Bergquist, 1961)

Corticella novae-zealandiae Bergquist 1961: 45, fig. 17
Corticellopsis novaezealandiae ; Bergquist 1968: 62, fig. 29, pl. 15 fig. C.

####### Material examined.

None.

####### Description

(from Bergquist 1961, 1968). Encrusting to massive sponge, smooth but uneven surface. Consistency firm. Colour white.

Skeleton: no data.

Spicules: calthrops, strongylasters, oxyasters.

Calthrops, occasionally with five or six cladi, 120–210 × 26–38 µm; “occasionally with dicho-modifications”; the 1968 illustration (Fig. 29) shows at least one proper short-shafted dichocalthrops.

Strongylasters, rather irregular in shape, with stunted rays, but not with a thickly silicified centre, 11 µm.

Oxyasters, 50–70 µm (5–7 rays).

####### Habitat.

Sublittoral fringe.

####### Distribution.

Rangitoto Island, northern New Zealand.

####### Remarks.

The drawings and photos of the asters are limited to the rather irregular strongylasters, whereas no illustrations are available of the oxyasters. It is assumed these were regular and unremarkable.

###### Calthropella Corticellopsis sp.

Figs 33A–C 

####### Material examined.

ZMA Por. 10525, Seychelles, Mahé, Cap Maçons & Anse de Forbans, 4.7667°S; 55.5167°E, 0–6 m, NIOP Expedition stat. 612, coll. R.W.M. Van Soest, nr. 612/20, 12 December 1992.

####### Description.

Cartilaginous crust on a piece of dead coral, surface smooth, size 2×1×0.5 cm. Greyish black alive, pale brown in alcohol.

Skeleton: with low spicular density, predominantly consisting of calthrops.

Spicules: Calthrops, euasters in two categories.

**Figure 33.**
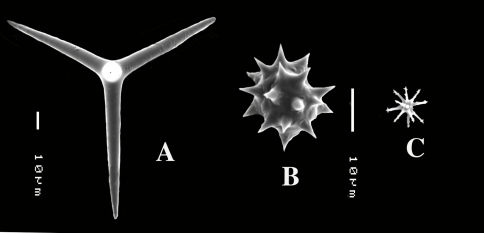
Calthropella (Corticellopsis) sp., ZMA Por. 10525, from Mahé, Seychelles, **A** calthrops **B** spheraster **C** oxyaster.

Calthrops (Fig. 33A) small, often with one cladus longer than the other (short shafted triaene), 30–*99.2*–195 × 4–*8.4*–11 µm, cladomes 46–*128.7*–255 µm.

Oxyspherasters (Fig. 33B), multirayed, rare, 9–*12.3*–18 µm.

Small oxyasters (Fig. 33C), approx. 10 rayed, without center, appear faintly tylaster-like, 6–*7.6*–11 µm.

####### Habitat.

Encrusting dead corals in shallow water.

####### Distribution.

Known only from a single locality south of Victoria, Mahé.

####### Remarks.

We refrain from naming this material due to the rarity of its microscleres. By its small sized calthrops this is a distinct species, as no matching descriptions appear in literature dealing with Calthropella s.l. The oxyspherasters were quite rare, and they could not be detected in any of the thick sections, whereas the small oxyasters were only marginally less rare, so there is a possibility that they are not proper to this sponge. The cartilaginous nature and low spicular density of the specimen reminded of Dercitus rather than of Calthropella, but we did not find sanidasters.

######## Nominal Calthropella species excluded from the genus

#### Nominal Calthropella species excluded from the genus

##### Calthropella digitata

Pulitzer-Finali, 1993

Calthropella digitata Pulitzer-Finali 1993: 252, Figs 2–3.

###### Material examined.

None. Holotype MSNG 48290

###### Description.

Branching mass of 5×3 cm. Confused skeleton in which giant oxeas (2700 × 9 µm) predominate. Next to this there are large dichotriaenes with cladomes of 400–550 µm wide and similar sized rhabdomes of 400–650 × 35–45 µm. Spherasters of 8–14 µm comprise the microscleres.

###### Habitat.

Deep water, 120 m.

###### Distribution.

North Kenya Banks, E Africa.

###### Remarks.

This is in all probability not a Calthropella as it has a dense skeleton with confusedly arranged oxeas. This species is best assigned to Stelletta s.l..

## Discussion

From the above presented data a close relationship of Dercitus s.l. and Calthropella s.l. has been made plausible, notwithstanding a clear separation in microsclere complement, shape and texture. The assignment of these two genera to different astrophorid families appears unnecessary, because Dercitus, although lacking euasters, does not possess clear pachastrellid streptasters other than acanthomicrorhabd-like sanidasters. Several Calthropella species, although possessing euasters, have heavily silicified microscleres similar to some Dercitus species. These microspined silica bodies can be morphologically derived from euasters (e.g. those of Calthropella (Calthropella) or from microrhabds (those of Calthropella (Pachataxa). The ovoid microspined microscleres of Dercitus (Halinastra) arubensis sp. n. appear to bridge the gap between Dercitus (Halinastra) and Calthropella (Pachataxa).

Assuming that the similarities will be found to be homologous and that the two considered groups will be found to be closely related, family assignment to one of the currently recognized families is not unequivocal. Calthropella at present is a member of the small family Calthropellidae, which – with the above proposals of generic synonymy – is reduced to virtually a single genus (Pachastrissa, Pachataxa and Corticellopsis are junior synonyms, Chelotropella is an ancorinid because of a radiating skeleton of long-shafted triaenes). Dercitus s.l. could be assigned as a second genus to Calthropellidae, but defining such a modified family is problematic in view of the fact that Triptolemma shares similar skeletal structure but has undoubted pachastrellid characters in the form of genuine streptasters (amphiasters, spirasters). Similarity of Triptolemma to Dercitus s.l. appears to be considerable because next to (dicho-)mesotriaene megascleres this genus also has calthrops and acanthomicrorhabds. Some species of the type genus of Pachastrellidae, Pachastrella, although having a skeleton with structural oxeas, appear close again to Triptolemma in sharing calthrops, dichocalthrops, mesotriaenes, streptasters and acanthomicrorhabds. This could mean that the above observed similarities are homoplastic rather than homologous, demonstrating independently acquired losses possibly induced by adaptations to the crevice-agglutinating-endolithic habitat. Thus, our results seem ambiguous for eventual changes in the classification. We will await further independent evidence for a revised classification of astrophorid genera.

### Further diversity

Including the three unnamed species described above, there appear to be at least seven records of Dercitus and Calthropella that likely concern distinct species in need of further description and differentiation before they can be properly named:

– Dercitus (Stoeba) sp. from Bonaire

– Dercitus (Stoeba) sp. from Madagascar

– Dercitus (Stoeba) sp. aff. *plicatus* sensu Sollas 1902 from Malaysia

– Dercitus (Stoeba) sp. aff. *plicatus* sensu Calcinai et al. 2000 from Maldives

– Dercitus (Halinastra) sp. aff. *luteus* from offshore Brazil

– Calthropella (Calthropella) sp. aff. *geodioides* var. from Ambon

– Calthropella (Corticellopsis) sp. from Seychelles

On the other hand, there are at least six species suspected to be junior synonyms:

– Dercitus (Stoeba) extensus with possible synonym Dercitus (Stoeba) pauper,

– Dercitus (Stoeba) plicatus with two possible synonyms Dercitus (Stoeba) lesinensis and Dercitus (Stoeba) dissimilis,

– Calthropella (Calthropella) geodioides with possible synonym Calthropella (Calthropella) simplex,

– Calthropella (Calthropella) pathologica with possible synonym Calthropella (Calthropella) inopinata

– Calthropella (Corticellopsis) stelligera with possible synonym Calthropella (Corticellopsis) recondita.

A further two records (Dercitus (Dercitus) bucklandi sensu Teerling 1975 from the Western Gulf of Mexico, and Dercitus sp. sensu Rützler et al. 2000 from Belize) await description and assignment to subgenus.

### Distribution patterns

**Figure 34.**
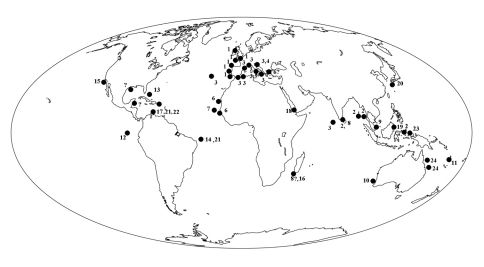
Distribution of species of the genus Dercitus. Black dots indicate approximate localities from which species were reported. **1** = Dercitus (Dercitus) bucklandi **2** = Dercitus (Stoeba) simplex **3** = Dercitus (Stoeba) plicatus **4** = Dercitus (Stoeba) lesinensis **5** = Dercitus (Stoeba) dissimilis **6** = Dercitus (Stoeba) senegalensis sp. n. **7** = Dercitus (Stoeba) verdensis sp. n. **8** = Dercitus (Stoeba) extensus **9** = Dercitus (Stoeba) pauper **10** = Dercitus (Stoeba) occultus **11** = Dercitus (Stoeba) fijiensis sp. n. **12** = Dercitus (Stoeba) reptans **13** = Dercitus (Stoeba) bahamensis sp. n. **14** = Dercitus (Stoeba) latex **15** = Dercitus (Stoeba) syrmatitus **16** = Dercitus spec. Madagascar **17** = Dercitus spec. Bonaire **18** = Dercitus (Halinastra) exostoticus **19** = Dercitus (Halinastra) berau sp. n. **20** = Dercitus (Halinastra) japnonensis sp. n. **21** = Dercitus (Halinastra) luteus **22** = Dercitus (Halinastra) arubensis sp. n. **23** = Dercitus (Halinastra) sibogae sp. n. **24** = Dercitus (Stoeba) xanthus. Question marks indicate position of unspecified records of Dercitus samples.

Previous analysis of generic distributions of Demospongiae (Van Soest 1994) indicated that a circum-global temperate and warm water occurrence is the most common pattern. Dercitus and Calthropella are probably following this pattern closely. Dercitus s.l. (Fig. 34) occurs in a circumglobal belt including the following provinces and ecoregions in the sense of Spalding et al. 2007: Celtic Seas (Marine Ecoregion of the Temperate Northern Atlantic Province), Lusitanian Province, Mediterranean Sea, Warm Temperate North West Atlantic, Warm Temperate Northwest Pacific, Warm Temperate North East Pacific, the whole of the tropical Atlantic, Western Indo-Pacific, Central Indo Pacific (including the Northeast Australian Shelf Province) and Tropical Eastern Pacific Realms. It is so far almost lacking from the southern warm temperate provinces, except for the Shark Bay Marine Ecoregion on the West Australian shelf. Most species occur in shallow-water, but a few are reported from below 100 m (Dercitus (Stoeba) bahamensis sp. n., Dercitus (Halinastra) sibogae sp. n., Dercitus (Halinastra) japonensis sp. n.). The subgenus Dercitus (Dercitus) has an oddly limited distribution in the Celtic Seas and South European Atlantic shelf marine ecoregions. Subgenus Stoeba is the most widespread, occurring over almost all provinces mentioned above. Subgenus Halinastra, although less common, likewise is represented in provinces encircling the globe. It is lacking so far in the Eastern Pacific and Eastern Atlantic provinces.

**Figure 35.**
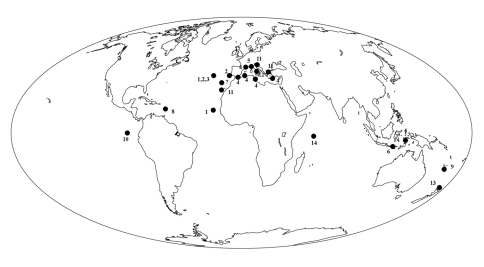
Distribution of species of the genus Calthropella. Black dots indicate approximate localities from which species were reported. **1** = Calthropella (Calthropella) simplex **2** = Calthropella (Calthropella) geodioides **3** = Calthropella (Calthropella) durissima **4** = Calthropella (Calthropella) pathologica **5** = Calthropella (Calthropella) inopinata **6** = Calthropella (Calthropella) xavierae sp. n. **7** = Calthropella (Calthropella) spec. Ambon **8** = Calthropella (Pachataxa) lithistina **9** = Calthropella (Pachataxa) enigmatica **10** = Calthropella (Pachataxa) pyrifera sp. n. **11** = Calthropella (Corticellopsis) stelligera **12** = Calthropella (Corticellopsis) recondita **13** = Calthropella (Corticellopsis) novaezealandiae **14** = Calthropella (Corticellopsis) spec. Seychelles. Question mark indicates position of an unspecified record of Calthropella sample.

Calthropella s.l. (Fig. 35) is less speciose, so far, but is also circumglobally distributed, occurring in most provinces that also contain Dercitus species. However, in the northern hemisphere the genus is lacking from provinces and regions north of the tropical provinces: it is not found in the Celtic Seas, the Gulf of Mexico and the Southern Californian Bight region. However, in the regions south of the tropics one species occurs in the Northeastern New Zealand province. Most species, excepting those of subgenus Corticellopsis, are found in deep-water and are rarely occurring above 100 m. Subgenera Calthropella (Calthropella) and Calthropella (Corticellopsis) so far are not known from the Caribbean. Subgenus Pachataxa is lacking from the Eastern Atlantic and Mediterranean. It is likely that these distribution patterns will turn out to be less disjunct when more information on the distribution of members of this genus is collected.

## Supplementary Material

XML Treatment for Dercitus

XML Treatment for Dercitus loricatus

XML Treatment for Stoeba natalensis

XML Treatment for Calthropella

XML Treatment for Calthropella digitata
